# Increased HIV Incidence in Men Who Have Sex with Men Despite High Levels of ART-Induced Viral Suppression: Analysis of an Extensively Documented Epidemic

**DOI:** 10.1371/journal.pone.0055312

**Published:** 2013-02-15

**Authors:** Andrew N. Phillips, Valentina Cambiano, Fumiyo Nakagawa, Alison E. Brown, Fiona Lampe, Alison Rodger, Alec Miners, Jonathan Elford, Graham Hart, Anne M. Johnson, Jens Lundgren, Valerie C. Delpech

**Affiliations:** 1 Research Department of Infection & Population Health, UCL, London, United Kingdom; 2 Health Protection Agency, London, United Kingdom; 3 London School of Hygiene and Tropical Medicine, London, United Kingdom; 4 City University, London, United Kingdom; 5 Copenhagen University Hospital/Rigshospitalet, and University of Copenhagen, Copenhagen, Denmark; Institut Pasteur of Shanghai, Chinese Academy of Sciences, China

## Abstract

**Background:**

There is interest in expanding ART to prevent HIV transmission, but in the group with the highest levels of ART use, men-who-have-sex-with-men (MSM), numbers of new infections diagnosed each year have not decreased as ARTcoverage has increased for reasons which remain unclear.

**Methods:**

We analysed data on the HIV-epidemic in MSM in the UK from a range of sources using an individual-based simulation model. Model runs using parameter sets found to result in good model fit were used to infer changes in HIV-incidence and risk behaviour.

**Results:**

HIV-incidence has increased (estimated mean incidence 0.30/100 person-years 1990–1997, 0.45/100 py 1998–2010), associated with a modest (26%) rise in condomless sex. We also explored counter-factual scenarios: had ART not been introduced, but the rise in condomless sex had still occurred, then incidence 2006–2010 was 68% higher; a policy of ART initiation in all diagnosed with HIV from 2001 resulted in 32% lower incidence; had levels of HIV testing been higher (68% tested/year instead of 25%) incidence was 25% lower; a combination of higher testing and ART at diagnosis resulted in 62% lower incidence; cessation of all condom use in 2000 resulted in a 424% increase in incidence. In 2010, we estimate that undiagnosed men, the majority in primary infection, accounted for 82% of new infections.

**Conclusion:**

A rise in HIV-incidence has occurred in MSM in the UK despite an only modest increase in levels of condomless sex and high coverage of ART. ART has almost certainly exerted a limiting effect on incidence. Much higher rates of HIV testing combined with initiation of ART at diagnosis would be likely to lead to substantial reductions in HIV incidence. Increased condom use should be promoted to avoid the erosion of the benefits of ART and to prevent other serious sexually transmitted infections.

## Introduction

Epidemics of HIV in men who have sex with men (MSM) started in the late 1970 s and early 1980 s and the numbers of new diagnoses continue to increase in several countries [1]–[7]. In the UK, for example, over 3000 MSM were diagnosed with HIV in 2010, the highest number since the start of the epidemic [1]. If we are to make informed choices on how to reduce new infections it is important to understand past trends in the epidemic and the factors which shaped them. Changes in self-reported condomless anal sex with persons of unknown or serodiscordant HIV status are clearly one key potential factor. Another potential factor is use of antiretroviral therapy (ART), which reduces transmission risk as well as reversing HIV progression [8]–[9]. The relative impact of these two factors on the MSM HIV epidemic are uncertain. Ecological analyses have observed correlations between ART use and trends in HIV diagnosis [10]–[11] (albeit one in the context of a mainly IDU epidemic) but these are difficult to interpret without an underlying model of transmission. There is great interest in the possibility of extending ART use in order to help to reduce HIV incidence [12]–[14], but a cautionary consideration is that the rise in incidence observed in MSM has occurred during a period in which ART use has expanded and the proportion of people with viral suppression has increased [1], [15]–[16]. Here we aim to use a comprehensive model of HIV transmission, progression and the effect of ART to reconstruct the HIV epidemic among MSM in the UK using comprehensive HIV surveillance data and behaviour data on self-reported condom use among MSM. The model aims to help us understand the relative influences of sexual risk behaviour change, rates of HIV testing, and ART-induced virologic suppression on HIV incidence over the past 15 years. While we focus on the UK our findings are likely to have broad implications for epidemics among MSM globally in resource rich settings.

## Methods

Here we describe the modelling briefly. Further details of the model are given in Supporting Information S1. Description of the current analyses are given in Supporting Information S2. We reconstruct sexual risk behaviour, HIV transmission, HIV progression and the effect of ART for the population of MSM in the UK from 1980–2010 using an individual-based stochastic computer simulation model which captures the key underlying mechanisms which determine these processes (model adapted from that used in ref 17, itself developed from models used in refs 16, 18–19]. We assume all transmission takes place via condomless anal sex and sexual risk behaviour was modelled as the number of short term (e.g. casual) and long term condomless sex partners which, as for all variables modelled, was updated in 3 month periods.

We consider a range of possible values (i.e. distributions) for the parameters that determine levels of condomless sex and, as for all other parameters, these are sampled from distributions of potential values that reflect uncertainty in the value. For example, it is assumed that a proportion of men (mean 0.5, defined by a beta distribution beta(7,7)) substantially reduce condomless sex with short term partners after HIV diagnosis (the proportion is much higher for long term partners). For each run of the simulation model we sample from each of these distributions to obtain a set of parameter values to be used and generate a full reconstructed epidemic scenario for 1980–2010. We then consider the parameter sets for runs that provide the closest fit to the observed data (as described in detail in Supporting Information S2 *)*. We ran the model 10,000 times in order to search for these best fitting parameter sets. Details of distributions for all parameters are given in Supporting Information S2. The sampling from distributions for the parameters relating to sexual risk behaviour and transmission rate means that we explore a range of possible scenarios, including ones in which the ratio of short to long term partners increases or decreases or the number of short term partners occurring in the same period as a man has a longer term partner (concurrency) increases or decreases.

In any given period, the probability of an uninfected person having a condomless sex partner who is infected with HIV depends on their number of partners and on the prevalence of HIV amongst partnerships formed by other men in the population, accounting for patterns of age mixing. The probability of transmission on exposure to an infected partner, depends on the viral load level of the partner (obtained by sampling from the distribution of viral load levels in partnerships formed by HIV infected people, accounting for age), the estimated risk of transmission at that viral load and presence of a concurrent sexually transmitted infection. We assume a low rate of transmission when the viral load is undetectable (although there is little direct evidence to support this, for anal sex) but, again, we consider a distribution of possible values. For people who have become infected with HIV the variables modelled include; primary infection (a period of raised infectivity of duration 3 months), viral load, CD4 count,, adherence to ART, risk of AIDS and death. Resistance acquisition and transmission is also incorporated, and its effects accounted for, although this is not a focus in the present paper. The model of progression of HIV and the effect of ART has been shown to provide a generally close fit to observed data relating to natural progression of HIV infection and the effect of ART [16]–[19]. Based on data from NATSAL [20], we assume that the population of MSM aged over 15 was approximately 500,000 in 1980 and, as for the UK as a whole, has increased in size by 10% up to 2010. We compared model outputs with a wide array of available data [1], [20]–[28].

## Results

**Figure 1:**
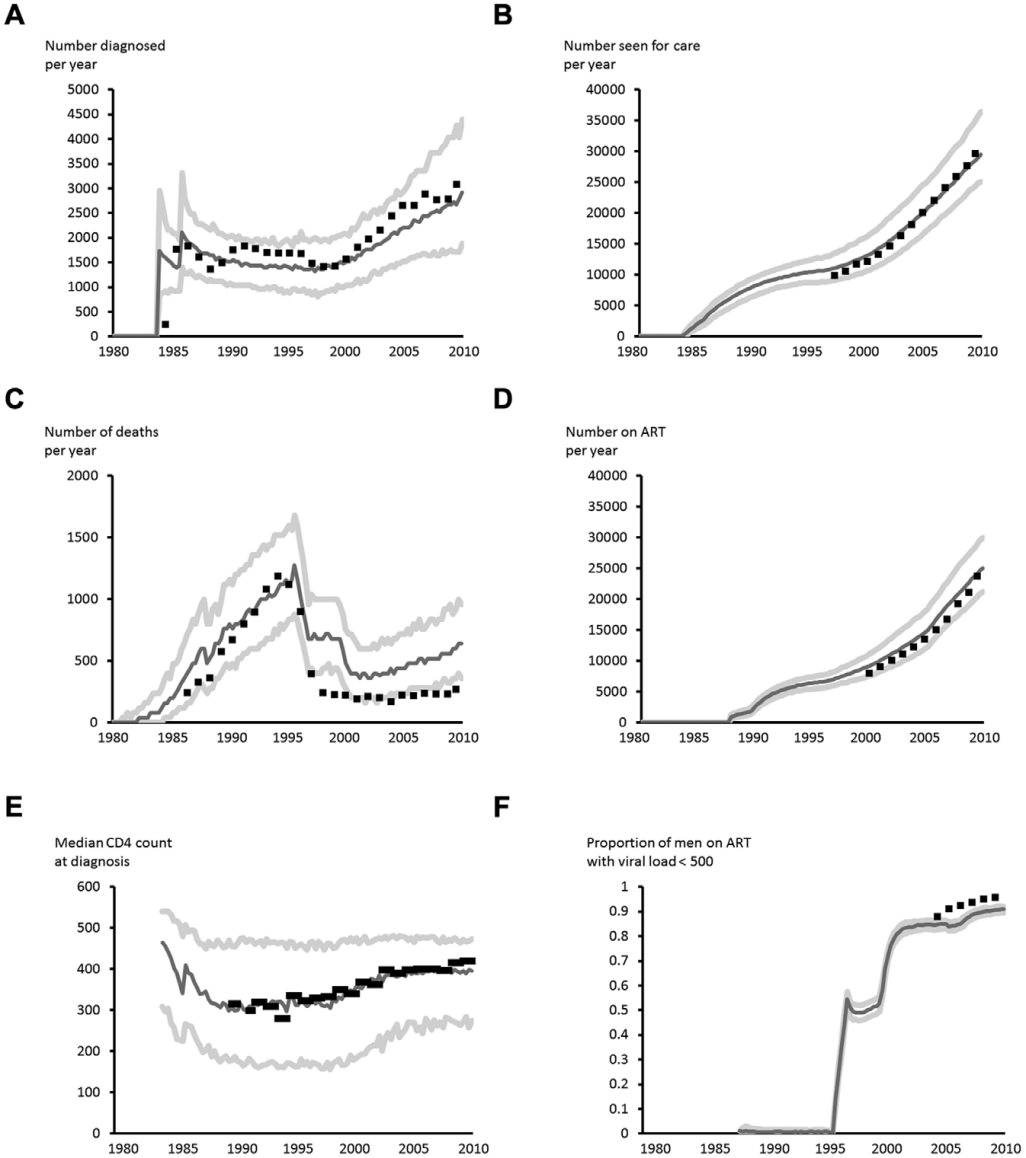
Comparison of model outputs with surveillance data. (a) Number of people diagnosed with HIV by year. Data points (black squares) from new HIV diagnoses database ^1^, (b) Number seen for HIV care by year. Data from SOPHID ^1^, (c) Number of deaths in people with HIV. Data from HPA death reporting system and Office of National Statistics data ^1^, (d) Number on ART. Observed data from SOPHID ^1^, (e) Median CD4 count at diagnosis. Observed data from HPA CD4 laboratory surveillance ^1^. (f) Proportion of men on ART with Viral load <500 copies/mL. Observed Data (black squares) from SOPHID ^10^. Model: median and 90% interval (dark and light grey lines, respectively). For details and further comparisons with data see Supporting Information S2.

**Figure 2:**
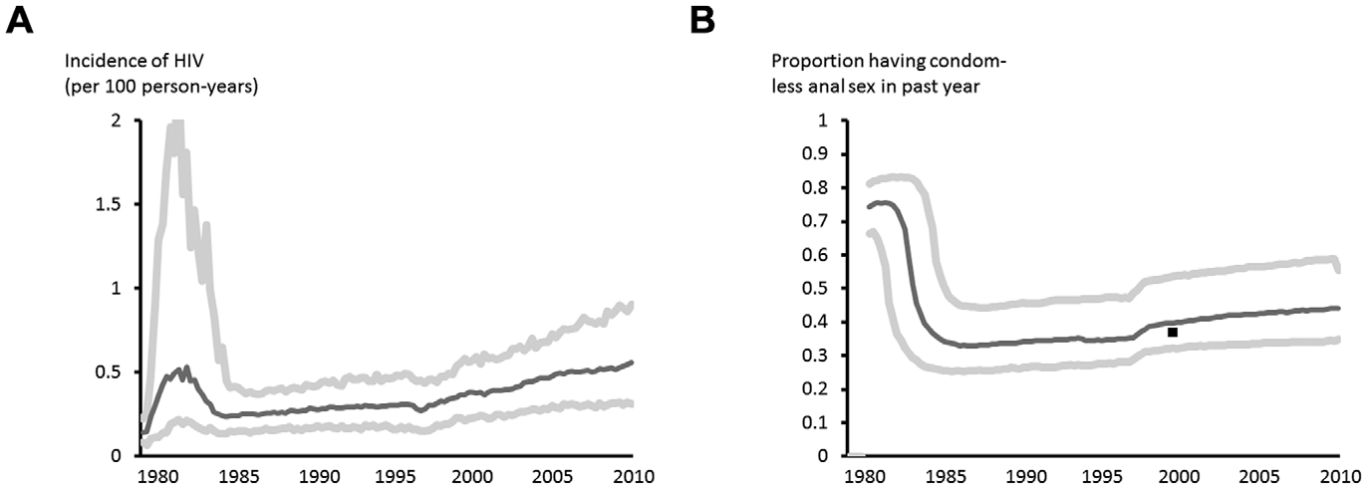
Estimated trends in HIV incidence and sexual behaviour. (A) HIV incidence, (B) proportion of men having more than one condomless sex partner in the past year.

The model outputs are generally consistent with a range of observed data collected as part of routine surveillance of HIV (Figure 1). A series of other model outputs are shown in Supporting Information S2. Implicit in this reconstruction is the underlying trend in incidence of new HIV infections and in sexual risk behaviour required to be consistent with the observed data. These are shown in figure 2, along with 90% uncertainty bounds reflecting parameter uncertainty.

The model suggests that after an initial period of high HIV incidence in the early 1980 s incidence declined in response to the decline in sexual risk behaviour. However, after the introduction of effective ART, the model shows an increase in sexual risk behaviour (from an estimated 35% of men having condomless anal sex with a partner of unknown or negative HIV status in the past year to 44% in 2010; a 26% increase) and this is associated with a rise in incidence, from a mean 0.30 per 100 person years from 1990–1997 to 0.45 from 1998–2010; p<0.0001. For the most recent five year period (2006–2010) the mean HIV incidence was 0.53. The breakdown of the source of new infections in 2010 according to the diagnosis, primary infection and ART status was calculated. Median proportions with 90% uncertainty bounds are: undiagnosed primary 0.48 (0.34–0.62), undiagnosed not primary 0.34 (0.22–0.46), diagnosed ART naive 0.10 (0.04–0.19), diagnosed ART experienced 0.07 (0.02–0.17). This indicates that a very high proportion of new infections derive from men who are undiagnosed, particularly men in primary HIV infection.

**Figure 3:**
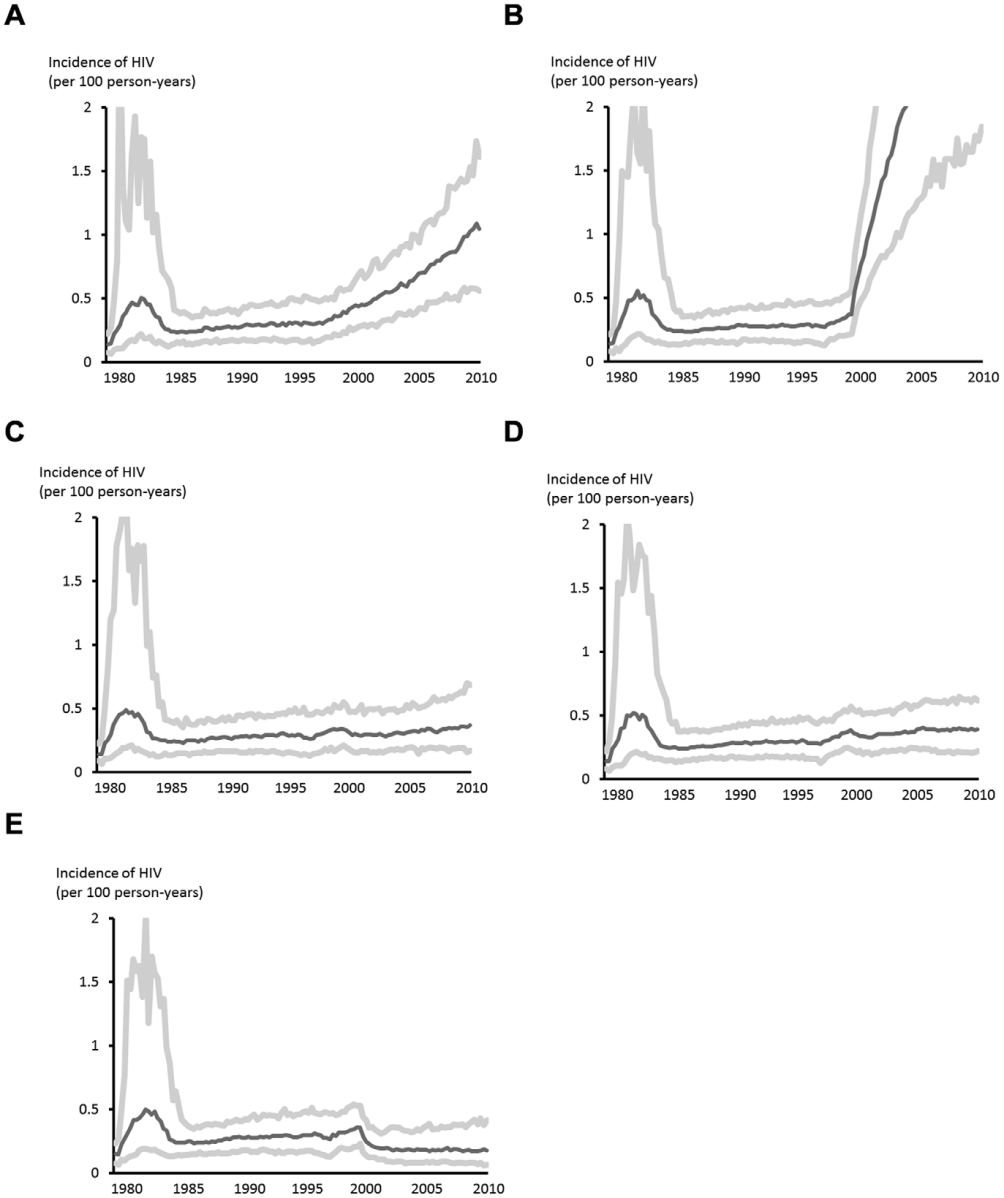
Reconstruction of incidence for counter-factual situations; (a) a scenario in which ART was never introduced, but patterns of sexual risk behaviour changes still occurred, (b) a scenario in which all condom use ceased in 2000, but with levels of anal sex as observed, (c) a scenario in which ART was provided at diagnosis from 2000, (d) a scenario in which testing rates increased (such that the proportion testing in the past year was 68% in 2010 compared with 25% as modelled for the actual incidence), and (e) a scenario of both higher testing and ART at diagnosis.

This detailed characterization of the course of the epidemic with a mechanistic model allows us to explore counter-factual scenarios which can help us understand the separate influences of HIV testing, ART use and of condom use on HIV incidence (Table 1). The model was re-run for a scenario in which ART was never introduced, but patterns of testing and sexual risk behaviour change were left unchanged (i.e. with an increase in sexual risk behaviour after 1998 - this is done in order to separate the direct effect of ART on incidence via lower viral load from its effect on increased condomless sex). Figure 3a shows the HIV incidence curve in this scenario, which shows a substantially greater increase in incidence than has been observed for the 2006–2010 period (0.53 to 0.89/100 pyrs in 2006–2010, a 68% increase (95% confidence interval 62%–74%; p<0.0001).

**Table 1:** Summary of estimated difference in HIV incidence according to counter-factual scenarios.

Scenario	Mean incidence 2006–10 (/100 prs)	% difference(vs actual)	95% confidence interval	p-value
Actual	0.53	–	–	–
No ART*	0.89	+68%	+62%–+74%	p<0.0001
No condoms**	2.78	+425%	+406%–+442%	p<0.0001
ART at diagnosis***	0.36	−32%	−27%–−37%	p<0.0001
Higher test rate****	0.40	−25%	−20%–−28%	p<0.0001
Higher test rate and ART at diagnosis*****	0.20	−62%	−58%–−66%	p<0.0001

* scenario in which no ART was introduced, but sexual risk behaviour change still occurred (this is in order to separate the direct effect of ART on incidence via lower viral load from its effect on increased condomless sex).

** scenario in which in 2000 all condom use had ceased but levels of anal sex remained the same. This was done by assuming that levels of sexual risk behaviour increase such that the proportion of men with a condomless sexual partner is set to the reported levels of sex, including condom-protected sex.

*** scenario in which policy from 2000 was to initiate ART in all people with diagnosed HIV.

**** scenario in which testing rates were much higher from 2000, such that by 2010 68% of all men were tested each year (49% per 3 months, with targeting of men having condomless sex in the past 6 months) compared with the figure of 25% used in modelling (6% per 3 months).

***** scenario with both higher rates of testing and ART initiation at diagnosis.

Next, we considered what would be the predicted effect on HIV incidence if in 2000 all condom use had ceased but changes in levels of anal sex and ART coverage in those diagnosed remained unchanged. This was done by assuming that levels of sexual risk behaviour increase such that the proportion of men having condomless anal sex in the past year is set closer to the reported levels of (condom-protected plus condomless) anal sex [20] such that 63% have condomless sex in past year. Here we see very large increases in incidence. We also considered what would have been the pattern of HIV incidence had ART, from 2001 onwards, been provided to all people with diagnosed HIV, with all else having remained the same, resulting in a predicted lower incidence for 2006–2010 (0.36/100 pyrs; a 32% (95% CI 27%–37%; p<0.0001) reduced incidence. Had rates of testing been greater then there would be expected to have been a lower incidence as a result of the fact that more people eligible for ART would be treated, and that diagnosis leads a proportion of men reducing or eliminating condomless sex with men of unknown or negative HV status. We estimated that had testing rates been considerably higher (such that by 2010 68% of all men were tested each year and 49% tested in any one 3 month period, with targeting of men having condomless sex in the past 6 months, compared with the figures of 25% and 6%, respectively, in the observed scenario), then incidence would have been 0.40/110 pyrs, a 25% (95% CI 20%–28%; p<0.0001) reduction. Finally, with both higher rates of testing and ART initiation at diagnosis predicted incidence was 0.20/100 pyrs; a 62% (95% CI; 58%–66%; p<0.0001) reduction.

## Discussion

This reconstruction of an HIV epidemic in MSM based upon multiple rich data sources allows us to understand the influences that condom use, HIV testing and ART have had on HIV incidence at a population level. Our analysis suggests that use of ART, even under a policy of ART initiation only when the CD4 count is below 200 or, latterly, below 350 and in the context of only a modest rate of HIV diagnosis, has had an appreciable impact on HIV incidence in the UK, resulting in most new infections being from people who are yet to be diagnosed. We estimate that incidence would have been 68% higher than that actually observed had ART not been introduced (but sexual risk behaviour changes remaining unchanged) and more than five times higher than the observed had "safer" sex messages been ignored and condom use ceased.

Our study throws light on the apparent paradoxical increase in HIV incidence in MSM epidemics over a period in which ART coverage and viral suppression has been increasing. Our analysis suggests it is the counter-effect of concomitant increases in condomless sex amongst MSM as a whole that has resulted in a net increase in incidence; the model did not fit the data unless we assume such a rise. There is direct evidence of increases in condom less sex due to increases in other STIs as well as from self-report survey data [21], [23]–[27]. A recent paper based on the MSM epidemic in the Netherlands reached a similar conclusion [6]. It is noteworthy that only modest increases in condomless sex are enough to overcome the beneficial effects of ART, highlighting the vulnerability of any new prevention initiative, such as ART initiation at HIV diagnosis, if it leads to increases in condomless sex. While our modelling and empirical evidence strongly suggest that increases in the number of men having condomless sex are the key underlying reason for the increase in incidence we should consider other possible explanations. It might be suggested that this could be accounted for by increases in other sexually transmitted infections which facilitate HIV transmission, but any such increases are likely to be themselves the result of increases in condomless sex. Our model takes account of the fact that presence of such STIs faciitates transmission risk but does not explicitly model transmission of the other STIs. Other explanations that seem unlikely are that HIV has become more infectious over time or that transmission through oral sex is significant and has increased over time. Our model does not inform us of the transmission risk with oral sex.

A key implication of our results is that extension of ART coverage, by increasing rates of diagnosis and with initiation of ART at higher CD4 counts, is likely to have an appreciable effect on reducing HIV incidence in MSM epidemics, so long as this is not accompanied by further general increases in condom less anal sex in the MSM population. Our model allowed us to explore the counterfactual scenario in which from 2001 there was a policy of ART initiation of all people with HIV diagnosed, and it was predicted that this would have resulted in 32% lower HIV incidence in the years 2006–2010 than was estimated to actually be the case. This was based on the key assumption that introduction of this policy would not have resulted in any additional increases in condomless sex ("risk compensation"), either in the individuals treated or the general population. On an individual level, there is thus far no evidence that ART initiation leads to increases in sexual risk behaviour (i.e. greater likelihood of having condomless sex) but there is a clear possibility that this might be the case in future as the effects of ART on infectivity become more widely known [8], [9], [29]. There could also be a continued population level effect such that with a greater feeling of HIV as a benign and manageable condition, condomless sex generally increases further. However, it does not seem likely that a decision whether to use a condom would be directly dependent on policy on HIV testing and earlier ART initiation, so introduction of such a policy should not in itself have a negative effect on condom use.

Thus, we consider that key public health policy implication of this work is the need to promote frequent HIV testing, and to offer ART for the purposes of reduction in infectivity. Such an offer would involve an explanation that plasma HIV suppression with ART is likely to be associated with markedly reduced infectivity, although this has not been directly shown in couple studies in MSM (while there is little reason to suggest that this will not be the case, the exact magnitude of the effect could well be different and ongoing studies of this are critical - our analysis does not in itself provide new evidence for the effect of ART at an individual level) [30]. The offer of ART to people with CD4 count above 350/mm^3^ should in our view be accompanied by an explanation of the fact that health risks associated with ART initiation have not been reliably shown in a randomized trial to be outweighed by the benefits, although trials are ongoing [31]. This is the position adopted in the British HIV Association guidelines, although some guidelines currently recommend initiation of ART in people with high CD4 count based on observational studies, arguments for a compelling rationale, and extrapolation from existing trials [32], [33]. If shown to have individual health benefits, a policy of initiation of ART in all people with diagnosed HIV could be cost-effective, despite the low absolute risk of clinical disease in people with high CD4 count, due to the effect in reducing HIV incidence, but this should be evaluated formally in models that closely fit to observed data for a given specific setting. At first sight, our finding - consistent with data and some other models [34]–[40] - of a high proportion of new infections emanating from men who are in primary infection, suggests a limit to the extent of the potential impact of use of ART on HIV incidence. As Koopman has pointed out [37], the key role of primary infection arises not only due to the high levels of infectiousness in this period, but because the variation over time in partnership formation rate means that people tend to get infected in a period in which the condomless sex partner acquisition rate is higher and thus the probability of onward transmission will tend to be higher soon after infection than later. This results in periods in which the effective reproductive number for primary infection is above 1, so "outbreaks" of primary infection are seen. Relatively few men are diagnosed with HIV at this early stage but there is potential for improvement [41] and this requires close attention. However, our modeling predicts that HIV incidence would be reduced substantially with a substantial increase in HIV testing (both in coverage of who is tested and frequency of testing in those tested) and immediate ART initiation in those diagnosed, despite the fact that diagnosis of HIV and treatment initiation cannot be achieved within the period of primary infection. This is likely because many chains of transmission are likely to contain links which involve transmission from people who have been infected for many months or years and these links can feasibly be broken with a policy of high levels of testing and immediate ART initiation, thus reducing the frequency of outbreaks of primary infection.

A second important message is the fact that promotion of condom use remains a critically important and effective element of prevention policies as it is undoubtedly acting to prevent much more dramatic increases in incidence. There is known to be selective condom use according to known or assumed partner HIV serostatus [42]. The message regardiing condom use may need to become more nuanced as evidence concerning the effectiveness of virally suppressive ART grows [8], [9], [30]. Under certain strict conditions regarding viral suppression and lack of current STIs, the risk that HIV will be transmitted to a negative person through condomless sex could be lower than that with use of condoms, given the non-negligible risks of failure to use a condom effectively [43]. However, in general, condom use remains critical to control of HIV epidemics and to reduce risk of acquisition of other sexually transmitted infections such as hepatitis C virus.

Recent modelling of HIV incidence in MSM based on a Bayesian evidence synthesis approach also found evidence of increases in incidence between 2002–2007 [44]. In order to help to predict future trends in incidence it will be helpful to obtain serial annual data on condomless anal sex on representative samples of MSM. Currently, the NATSAL data are the only data based on a random sample, but are only available every 10 years and numbers of MSM included are small. Interpretation of trends from the Gay Men's Sex Surveys [20] is hampered by the opportunistic self-selecting sampling approach but nevertheless the data are of critical importance in the absence of other sources, as are the series of studies in gyms and bars and online samples [23]–[26]. Other models of MSM epidemics [45]–[47] have evaluated the potential effect of ART on transmission.

In conclusion, it seems likely that modest increases in condomless sex in the era of effective ART in the UK have resulted in an increase in HIV incidence in MSM, but that the effects of ART in reducing infectivity have substantially attenuated this effect. More frequent HIV testing and better penetration of regular testing to all MSM is critical. This would be likely to be more effective if clinical practice moves towards ART being prescribed at diagnosis. The promotion of condom use among negative as well as HIV positive MSM remains vital to ensure the benefits of ART in reducing transmission of HIV are not undermined.

## Supporting Information

Supporting Information S1 **Model Details.**
(DOC)

Supporting Information S2 **Supplementary Analysis Methods and Results.**
(DOC)
