# Supplementary material for: Increased HIV Incidence in Men Who Have Sex with Men Despite High Levels of ART-Induced Viral Suppression: Analysis of an Extensively Documented Epidemic
Source: PLoS One. 2013 Feb 15;8(2):e55312. doi: 10.1371/journal.pone.0055312 (PMC3574102; doi:10.1371/journal.pone.0055312)
Supplement: Supporting Information S1 — Model Details. (DOC) [file pone.0055312.s001.doc]

**Supporting information S1**

**Increased HIV incidence in men who have sex with men despite high levels of ART-induced viral suppression: analysis of an extensively documented epidemic**

**Model Details**

# Demography, risk behaviour and transmission of HIV

## Demographic model

### Age distribution and death rates for general population

The model runs for 50 years from 1980, with variables updated in 3 month periods (although here we focus only on runs to 2010). Three months is an arbitrary choice of time period which allows sufficient sensitivity in capturing changes over time and is also computationally feasible. Each run of the simulation program creates 130,000 simulated people, although only ~ 50,000 are alive and aged 15 in 1980. Due to decreasing death rates over time the population size increases over time from 1980-2010 by approximately 10%. at any point in time.

Age specific death rates for uninfected people (based on UK male death rates in 2008) are as follows:

| Age group | Annual death rate |
| --- | --- |
| 15-19 | 0.00043 |
| 20-24 | 0.00065 |
| 25-34 | 0.00087 |
| 35-44 | 0.00159 |
| 45-54 | 0.00345 |
| 55-64 | 0.00860 |
| 65-74 | 0.02200 |
| 75-84 | 0.06140 |
| >85 | 0.16230 |

These rates are modified as follows to account for the decrease in death rates over time.

death_rate at date *d* = death_rate x 1.015(2008 - *d*)

These death rates are modified by a factor 1.5 for smokers and by 0.75 for non-smokers (see part 2). This is due to the known effects of smoking on all-cause mortality(1).

The actual probability of dying in a 3 month period then is estimated as: 1 – *exp* (-0.25*annual_death_rate)

The initial age distribution is determined on the basis of the following distribution:

| Age group | Probability of being in age group in 1980* |
| --- | --- |
| -35-14 | 0.45 |
| 15-24 | 0.088 |
| 25-34 | 0.081 |
| 35-44 | 0.080 |
| 45-54 | 0.080 |
| 55-64 | 0.081 |
| 65-74 | 0.072 |
| 75-84 | 0.045 |
| >85 | 0.023 |

* the actual age of a person in a given group in 1980 is determined by sampling from a Uniform distribution

This distribution is chosen to be consistent with the male population of the UK.

Thus around half of simulated people have an age below 15 in 1980. Besides calendar year, the only variable that is modelled and updated up to reaching the age of 15 (when becoming potentially sexually active) is age itself. The “youngest” person in 1980 is age -35 (i.e. will be born in 2015 and reach age 15 in 2030, when the modelling period ends).

## Model of sexual risk behaviour and risk of HIV acquisition

Risk behaviour is characterized by two variables representing, respectively, the number of short term condomless sex partners and whether the man has a current longer term condomless sex partner in the 3 month period. The status of longer term partners is tracked over time (i.e. if they are infected, diagnosed, on ART, etc.). The rationale for explicitly considering short and long term partners is that we know that people have short term and long term partnerships and we wished to track long term partnerships so that the HIV status of the partner (as well as their diagnosis and ART status) could be modelled as this is an important potential source of infection.

### Determination of number of short term partners at period *t*

Numbers of short term partners in a given period was generated at random, according to which of four risk behaviour groups the person was in for this period (see also Table 1). Changes in the risk behaviour group from *t-1* to *t* were determined by transition probabilities between 4 groups: no short term condomless partners in 3 month period, 1 short term partner, 2-10 short term partners, and 10 or more short term partners.

Transition probabilities pija of moving from partner group i at *t-1* to partner group j at *t* are given by:

| pija = fij /(fgi1 + Σj=2-4 (f­ij. ra)) | for j=1 |
| --- | --- |
| pija = fij. ra /(fgi1 + Σj=2-4 (f­ij. ra)) | for j=2 - 4 |

where a = 1-10 for age groups 15-, 20-, 25-, 30-, 35-, 40-, 45-, 50-, 55-, 60-, respectively.
Values of f­ij and ra are given in Tables 1 and 2, respectively.

Values of ra are modified at time *t* by a factor 0.2 if the subject has a current AIDS defining disease and by a factor *i_ch_risk_diag* if the subject is diagnosed with HIV. *i_ch_risk_diag* is a non-time-varying individual-level varable determined on the basis of a parameter *ch_risk_diag* (distribution beta(5,1))which indicates the probability that an individual has a reduction in numbers of short term partners after HIV diagnosis (in which case ra is modified by a factor 0.1). In addition, there is a person-fixed modification factor. For a random 20% (distribution 0.40 x exp (Normal(0, 0.20) / 2) of men values of ra are modified by a factor 0.25,and for a further 15% (distribution 0.40 x exp (Normal(0, 0.20) / 2) of men by a factor 0.50, to reflect the fact that a proportion of people experience only very low sexual risk activity throughout their life.

Actual transitions between groups were determined by random sampling. For the first two groups the number of partners in the period is given (i.e. no short term partners, 1 short term partner, respectively). When a person was in the 2-9 short term partners group the number of partners was determined by sampling from Poisson(3.5) (distribution 3.5 x exp (Normal(0, 0.20)), and when the transition was to > 10 short term partners the number of partners was determined by sampling from Poisson(2) and multiplied by a factor called *swn* (distribution 7 x exp (Normal(0, 0.20)).

### Determination of having a longer term (condomless) partner at period *t*

Again, only condomless sex partnerships are modelled. Thus if a person has a longer term partner but condoms are used on all occasions of sexual intercourse then this is not counted as having a longer term partner. In 1980, before any population level reductions in risk behaviour, at each period men with no current longer term condomless sex partner have probability 0.40 (distribution 0.40 x exp (Normal(0, 0.20)) of starting having condomless sex with a longer term partner. This can be due to (re-)starting having condomless sex with an existing longer term partner or starting a new partnership which involves condomless sex.

At the time a longer term partnership is started, it is classified into 3 duration groups, each with a different tendency to endure. The percent of people in each group is dependent on age and is shown in Table 3. At time period, *t*, for people with a longer term partner, the probability of the partnership continuing is 0.75 if duration category is 1, is 0.95 if duration category is 2, and 0.98 if duration category is 3.

The parameter values relating to sexual behaviour are based on comparing model outputs to observed data (2-6).

### Sexual mixing and determination of number of short term partners who are HIV infected at time *t*

For each short term partner that a man has at time *t*, the probability that the partner is infected is calculated. This is dependent on the prevalence of HIV in the MSM population, taking consideration of age mixing in sexual partnerships. If the subject is of age group a, then for each short term partner the first step is to determining by random sampling the age group, a’, of the short term partner (for simplicity, all the subject's short term partners during period *t* are assumed to be in this same age group). The age mixing probabilities used to determine this are given by values in Table 1.

Then, for the given partner (of age group a’), the risk that the partner is infected is then given by

ha(t) = Σ a’ L1(*t-1*) / Σa’ L(*t-1*)

where Σ a’ is the sum over all subjects of age group a’, L1(*t-1*) is the number of short term partnership formed by people infected with HIV at time *t-1*, and L(*t-1*) is the number of short term partnership at time *t-1*.

Since we assume that all short term partners in period of time *t* are in this same age group, the total number of infected short term partners that the subject has at time *t*, L1(*t*), is then given by

L1(*t*) = Min ( Poisson (ha(t) x L(*t*) ) , L(*t*) )

### Determination of probability that a longer term partner is HIV infected at time *t*

E1(*t*) indicates whether the subject has a longer term (condomless sex) partner who is infected (E1(*t*) =1 if infected, else E1(*t*) = 0). A longer term partner at time *t* can be infected either because (i) a new longer term partnership has been formed and the partner was already infected, (ii) because a longer term partner at *t-1*, which has remained a longer term partner at time *t*, has become infected, or (iii) because an infected longer partner has remained as a longer term partner.

In the first scenario (i), where a new CLLT partnership has been formed and the partner was already HIV infected, it is assumed that in 50% of the cases the CLLT partner is the same partner he had before in his/her life and it is tracked whether he/she is HIV infected. In addition the probability that he is HIV infected depends on the HIV age and gender specific HIV prevalence.

In the second scenario (ii), a CLLT partner at t-1, which has remained a CLLT partner at time t, has become HIV infected. At each point in time first we randomly determine whether a CLLT partner is monogamous (in terms of condomless sex), based on the age and gender specific proportion of people who are monogamous (in a CLLT relationship and zero CLST ones). If this is the case, this CLLT partner can be infected only by the subject we track and the probability of transmission is based on the VL and gender, as described in section 1.3).

If the CLLT partner is not “monogamous”, the probability that he/she becomes infected is derived from the HIV incidence at t-1 for age group a (i.e. the same age group) and gender 1-g among those who have a CLLT partnership and at least one short term partner, ia,1-g(t-1).

E1(t) = 1 if a sampled random variable from Uniform(0,1) < ia,1-g(t-1), else E1(t) = 0

In order to maintain balance, for each gender, between the number of uninfected people with a CLLT partner who is HIV infected, and the number of HIV infected people with a CLLT partner who is uninfected, this incidence ia,1-g(t-1) is modified at time t dependent on the degree of balance at time t-1. The balance achieved is illustrated in Figure 11.

In the third scenario (iii), where a CLLT relationship with a partner infected with HIV has remained as such.

It is assumed that if a person had a CLLT relationship with a partner HIV infected at time t-1, and he/she is in a CLLT partnership at time t, the partner is the same and therefore he/she is HIV infected

If E1(t-1) = 1 and E(t) > 1 then we assign E1(t) = 1

### Determination of the risk of infection from a short term partner / assortativity

For each HIV infected short term partner his viral load group, v is obtained by sampling from the viral load distribution of the population of infected men, weighted by the number of partnerships formed. Thus we sample from Uniform(0,1), where the probability of the partner having viral load in group v is given by

Σv L1(*t-1*) / ΣL1(*t-1*)

where Σv is the sum over all HIV-infected subjects in viral load group v and Σ is the sum over all HIV-infected subjects.

Viral load groups are:

(1) < 2.7 log cps/mL

(2) 2.7-3.7 log cps/mL

(3) 3.7-4.7 log cps/mL

(4) 4.7-5.7 log cps/mL

(5) > 5.7 log cps/mL

(6) primary infection.

Once the viral load group, v, of the infected partner is determined, the probability, tv, of the subject being infected by the partner is sampled from the following distributions ing to:

t1 = Normal (0.002,0.000025), t2 = Normal (0.01,0.0025), t3 = Normal (0.03,0.0075), t4 = Normal (0.06,0.015), t5 = Normal (0.1,0.025), t6 = Normal (0.2,0.075). (The sampling from a distribution here is to reflect the variable number of sex acts).

These are based on ref (7). These probabilities are increased on average by 3-fold if the person has an existing STI (risk of a new STI in any one three month period is given by the number of short term condomless partners / 20 (or 1 if > 20 short term partners)) (8).

Realization of whether the subject is infected by each short term partner is determined by sampling from Uniform(0,1).

Choice of who is the partner for a man having a short term partner is based on the number of partnerships had by others in the population, so if for example one person has 10 short term partners in a 3 month period and another has 1 short term partner then, when sampling to select the viral load and primary infection status of the partnerships being formed in the population in the period, the viral load of the former contributes 10 times the observations to the viral load distribution.   In this sense, sexual mixing is assumed to be assortative. We consider it unlikely it could be less assortative than this.

### Determination of the risk of infection from a longer term partner

Infected longer term partners at time *t* are classified by whether they are in primary infection (if infection occurred at *t-1*), whether they are diagnosed with HIV, whether they are on ART, and whether their current viral load is < 2.7 cps/mL or not. The proportion of longer term partners with HIV who have HIV diagnosed at time *t*, pDe(*t*), is determined with reference to the difference, dDe(*t-1*), in the proportion of subjects with HIV who are diagnosed, TD(*t-1*) / T1(*t-1*), and pDe(*t-1*); i.e.

dDe(*t-1*) = TD(*t-1*) / T1(*t-1*) – pDe(*t-1*)

where TD(*t-1*) is the total number of subjects diagnosed with HIV at time *t-1*and T1(*t-1*) is the total number of subjects with HIV (diagnosed and undiagnosed) at time *t-1*.

If dDe(*t-1*) > 0 then for each subject the probability of the longer term partner being diagnosed is pDe(*t*) with the realization for the individual being determined by sampling from a Uniform (0,1) distribution

The proportion of those diagnosed who are on ART, and the proportion of those on ART who have viral load < 2.7 log cps/mL are determined in a similar manner. In this way the proportions diagnosed with HIV, on ART, and with current viral load is < 2.7 cps/mL are kept similar for the longer term partners as in the simulated subjects themselves.

Risk of infection from a longer term infected partner at time *t* is determined by probabilities gievn by sampling from Normal (0.2, 0.075) if the existing partner is in primary infection (i.e. infected at *t-1*), Normal (0.0001, 0.000025) if the existing partner has viral load < 2.7 cps/mL, and Normal (0.05, 0.0125) otherwise. The transmission rate is assumed to be higher from long term partners than short term partners (due to a higher number of sex acts) by a factor determined by sampling from the distribution 4 x exp (Normal(0, 0.20).

## Tables and figures referred to in Part 1

**Table 1**
Values of f­ij (values determining probability of transitioning between short term partner risk behaviour groups)

| Short term partners in period t-1 | Short term partners in period t | | | |
| --- | --- | --- | --- | --- |
|  | 0 | 1 | 2-9  Poisson mean 3.5* | > 10  Poisson mean 2 x 7* |
| 0 | 0.80 | 0.19 | 0.01 | 0.00 |
| 1 | 0.45 | 0.52 | 0.03 | 0.00003 |
| 2-9 | 0.15 | 0.35 | 0.50 | 0.0001 |
| > 10 | 0.03 | 0.07 | 0.20 | 0.70 |

* x exp (Normal(0, 0.30)

**Table 2**Values of ra (factor determining relative level of sexual risk activity by age)

| Age group (a=1,10) | ra |
| --- | --- |
| 15- | 1.20 |
| 20- | 1.50 |
| 25- | 1.80 |
| 30- | 2.00 |
| 35- | 1.00 |
| 40- | 0.75 |
| 45- | 0.60 |
| 50- | 0.50 |
| 55- | 0.35 |
| 60- | 0.25 |

**Table 3**Percent of newly formed longer term partnerships classified into each of three duration groups, each of which has a different tendency to endure (higher class, more durable)

| Age | 1 | 2 | 3 |
| --- | --- | --- | --- |
| 15-44 | 30% | 30% | 40% |
| 45-54 | 30% | 50% | 20% |
| 55-64 | 30% | 50% | 20% |

**Table 4**
The proportion of short term partnerships formed by subjects in age group a­s which are with men of age group ap

|  | Partner Age group (ap) | | | | |
| --- | --- | --- | --- | --- | --- |
| Subject Age group (as) | 15-24 | 25-34 | 35-44 | 45-54 | 55-64 |
| 15-24 | 0.56 | 0.26 | 0.10 | 0.09 | 0.06 |
| 25-34 | 0.35 | 0.54 | 0.38 | 0.22 | 0.09 |
| 35-44 | 0.06 | 0.16 | 0.32 | 0.29 | 0.09 |
| 45-54 | 0.02 | 0.03 | 0.18 | 0.27 | 0.23 |
| 55-64 | 0.01 | 0.01 | 0.02 | 0.13 | 0.53 |

**Figure 1**Total numbers of short term partners in one period (first quarter of 2000) by age group for subjects (black line) and expected numbers based on age mixing for sexual partners (grey line).

So, for example, the number of partners of men of age 15-25 matches the number of partners had by men of all ages with men of age 15-25, etc.


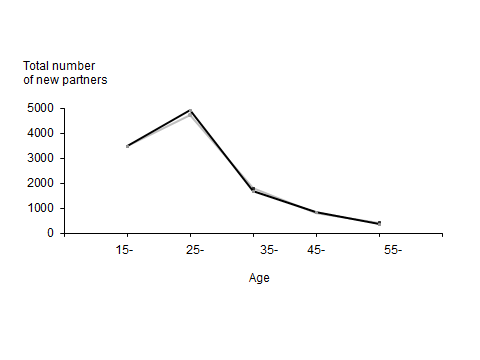


**Figure 2a** Proportion of men age 15-45 with at least one condomless sex partner in the past year by HIV status.

Early in the epidemic those infected are people who generally still within the phase of high risk activity that was the cause of their infection. As the epidemic matures, the average level of risk activity among those infected declines. This is due to the fact that the population of infected people is increasingly made up of people who were infected despite not having high risk activity (e.g. people infected by a longer term partner) and the fact that those who were infected during a period of high risk activity will tend to have reduced levels of sexual activity due to natural variability over time and reductions in risk activity with age.


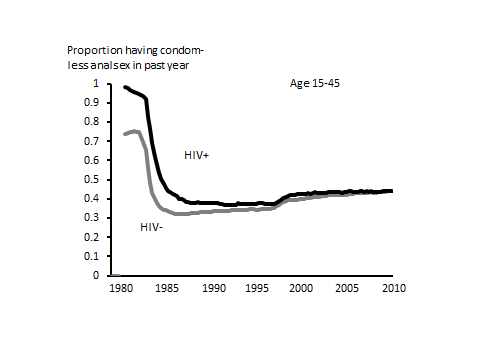


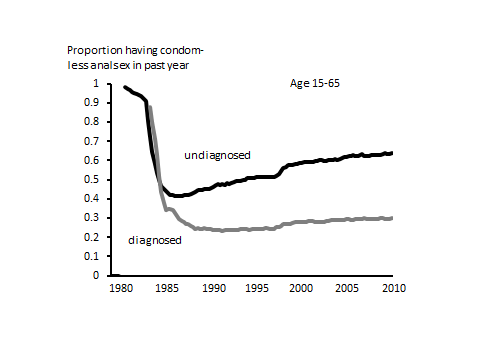
 **Figure 2b**
Proportion of HIV-infected men (age 15-65) with at least one condomless sex partner in the past year by diagnosis status.

# Untreated HIV infection

## Diagnosis of HIV

Individuals will only be diagnosed post-1984 (which is when HIV testing starts in the model).

The probability of someone testing for HIV in any 3 month period depends on their situation:

| Situation | Probability of HIV testing (per 3 month period) |
| --- | --- |
| Calendar year >= 1995 and currently in primary infection | 0.2 |
| Has an AIDS defining condition | 0.9 |
| Has had TB in the last 3 months, but not an AIDS defining condition | 0.5 |
| Has had a CDC B symptom in the last 3 months, but not TB nor an AIDS defining condition | 0.15 |

## Fixed parameters at time of infection

### Initial viral load

The initial viral load ‘set point’ (log copies/ml) is distributed as follows (i.e. Normal(4,0.5)):

The maximum viral load ‘set point’ is defined to be 6.5.

The viral load at the time of infection is equal to the initial viral load ‘set point’.

### Initial CD4 count

Initial CD4 count (cells/mm3) is determined on the square root scale. It also depends on the person’s viral load ‘set point’ as follows:

√ CD4 = 32 – (2 x viral load set point) + Normal(0,2)

Or equivalently, the CD4 count (not on square root scale) is approximately distributed as follows:

The minimum and maximum initial CD4 count is defined to be 324 and 1500 respectively.

### Shift to X4 virus

Initial virus is assumed to be R5-tropic.

Shift to presence of X4 virus is assumed to depend on viral load. The probability of shift in a 3-month period is given by 10v x 0.0000004, where v is the most recent viral load, and is thus distributed as follows:

**Comment:** This translates into a rate of 5% per year in a person with viral load 30,000 cps/mL and 16% per year in a person with 100,000 copies/mL, which are broadly consistent with observed data (9).

### Use of PCP Prophylaxis

In any given 3-month period, there is a 90% probability that a patient will be on PCP prophylaxis given they attend clinic visits and

- Their measured CD4 count is <200 cells/mm3
- or if they have ever been diagnosed with a CDC B symptom or with AIDS

### Subtypes

Currently in the MSM transmission model, the viral subtype is not specified; data to which it is fitted are mainly from Europe and so will reflect the subtypes in circulation – mainly B.

## Determination of viral load

### Changes in viral load

Viral load change (log copies/ml) from period *t-1* to *t* (i.e. in a 3 month period) is given by sampling from a normal distribution with standard deviation 0.05 and mean 0.02275:


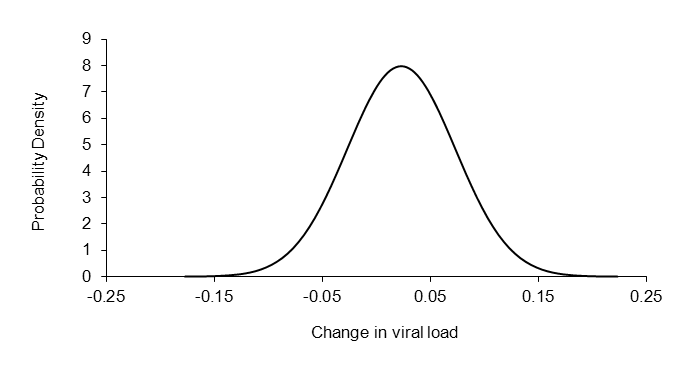


The maximum viral load is defined to be 6.5.

### Measured viral load

The measured viral load is given by the real viral load plus error (which is sampled from a normal distribution with mean 0 and standard deviation 0.2). This is to reflect the measurement error which arises each time a patient’s viral load is measured.

## Determination of CD4 count

### Changes in CD4 count

CD4 change (cells/mm3) from period *t-1* to *t* (i.e. in a 3 month period) is dependent on viral load at *t-1* and is given by sampling from a normal distribution with standard deviation 1.2 and mean as follows:

| Viral load at t-1 | Mean √ CD4 change (per 3 months) |
| --- | --- |
| < 3 | -0.027 |
| 3.0- | -0.072 |
| 3.5- | -0.135 |
| 4.0- | -0.180 |
| 4.5- | -0.450 |
| 5.0- | -0.900 |
| 5.5- | -1.800 |
| 6.0- | -2.250 |

In addition, the change in CD4 count is affected by current age as follows:

| Age | Additional √ CD4 change (per 3 months) |
| --- | --- |
| <20 | +0.15 |
| 20- | +0.09 |
| 25- | +0.06 |
| 30- | 0 |
| 35- | 0 |
| 40- | -0.06 |
| 45- | -0.09 |
| 50- | -0.15 |
| 60- | -0.20 |

People with X4 virus present experience an additional change in √ CD4 of -0.25.
The minimum CD4 is defined to be 0.

### Measured CD4 count

The measured CD4 count is given by the real CD4 plus error (on the square root scale, which is sampled from a normal distribution with mean 0 and standard deviation 2.0). This is to reflect the measurement error which arises each time a patient’s CD4 count is measured.

The model also includes the concept of a temporary drop in CD4 count close to the time of infection (to reflect the drop during primary infection), but only for measured CD4 counts and not for the underlying CD4 count as described in section 2.4.1. The drop is distributed ~N(-150,50) at the time of infection and is subtracted from the underlying CD4 count. Note that this drop in measured CD4 only relates to ART-naïve patients who have been diagnosed and are thus attending clinic (and therefore getting their CD4 counts measured).

The minimum measured CD4 is defined to be 0.

**Comment:** These estimates for viral load and CD4 count in sections 2.2 to 2.4 were selected in conjunction with other relevant parameter values to provide a good fit to the incubation period (to AIDS, death, CD4 thresholds) distribution, and to data on viral load and CD4 count which are given in the model fit. The estimates are derived based on the synthesis of evidence from natural history studies (10-17). Differences that have been found in initial viral load by sex, age and risk group are not currently incorporated in the model.

## CDC B / AIDS / Death

### Rate of occurrence of AIDS disease

The rate according to (most recent) CD4 count is as follows:

| CD4 count | Rate (per year) |  | CD4 count | Rate (per year) |
| --- | --- | --- | --- | --- |
| > 650 | 0.002 |  | 150 - 174 | 0.10 |
| 500 - 649 | 0.010 |  | 125 - 149 | 0.13 |
| 450 - 499 | 0.013 |  | 100 - 124 | 0.17 |
| 400 - 449 | 0.016 |  | 90 - 99 | 0.20 |
| 375 - 399 | 0.020 |  | 80 - 89 | 0.23 |
| 350 - 374 | 0.022 |  | 70 - 79 | 0.28 |
| 325 - 349 | 0.025 |  | 60 - 69 | 0.32 |
| 300 - 324 | 0.030 |  | 50 - 59 | 0.40 |
| 275 - 299 | 0.037 |  | 40 - 49 | 0.50 |
| 250 - 274 | 0.045 |  | 30 - 39 | 0.80 |
| 225 - 249 | 0.055 |  | 20 -29 | 1.10 |
| 200 - 224 | 0.065 |  | 10-19 | 1.80 |
| 175 - 199 | 0.080 |  | < 10 | 2.50 |

### Rate of occurrence of CDC Category B

The occurrence of CDC category B is taken to be 5-fold higher than the rate for AIDS (as in section 2.5.1).

20% of cases of these CDC B category B symptoms are assumed to be tuberculosis (TB).

### AIDS defining conditions (ADC)

In a given 3-month period, if a patient is diagnosed with an ADC, they are also diagnosed with AIDS (if they haven’t been diagnosed with AIDS previously).

### Rate of occurrence of HIV-related deaths

The occurrence of HIV-related deaths is taken to be 4-fold lower than the rate for AIDS (as in section 2.5.1).

**Comment:** The factor 4 was chosen to provide results consistent with observed data, including on the incubation period for death and the time from AIDS to death (in untreated people) (17-20).

#### Cause of deaths

The occurrence of deaths, which are explicitly NOT due to non-HIV causes, is closely related to CD4 count. Some of these deaths however, although related to CD4 count, will not be HIV-related (e.g. other cancers but not including liver deaths as these are modeled separately – see section 2.5.4.2). Therefore, of the CD4-related deaths, a proportion (15%) will be classified as non-HIV deaths, and the remaining 85% will be classified as HIV-related deaths.

#### Effect of hepatitis co-infection

It is assumed that liver death is closely related to CD4 count.

The rate of death from HIV is divided by 6 to obtain the rate of liver death for those co-infected with hepatitis B or C. These increases in the rate of liver deaths are based on data from observational studies (18;21-24).

## Independent effects on all rates, except death from non-HIV causes (i.e. AIDS / CDC B Symptoms / HIV-related deaths)

### Independent effect of viral load

Rates are independently affected by (most recent) viral load:

| Viral load (log) | Multiply rate by |
| --- | --- |
| < 3 | 0.2 |
| 3 - 3.99 | 0.3 |
| 4 - 4.49 | 0.6 |
| 4.5 - 4.99 | 0.9 |
| 5 - 5.49 | 1.2 |
| >= 5.5 | 1.6 |

### Independent effect of age

Rates increase with age. Multiply rate by (age/38)1.2.

For example:

| Age | Multiply rate by |
| --- | --- |
| 20 | 0.46 |
| 30 | 0.75 |
| 40 | 1.06 |
| 50 | 1.39 |

### Independent effect of PCP prophylaxis

If on PCP prophylaxis, multiply rates of AIDS and death from HIV (but not rates of CDC B Symptoms) by 0.8.

### Independent effect of being on ART

The rates depend on the number of drugs in the current regimen:

- Multiply rate by 0.9 if on single drug regimen
- Multiply rate by 0.85 if on 2 drug regimen
- Multiply rate by 0.8 if on 3 drug regimen to reflect that being on HAART has a positive effect on risk of AIDS and death, independent of latest CD4 count and viral load

**Comment:** These estimates for section 2.6 are broadly based on references (25-29).

### Independent effect of having TB

When an individual currently has TB or has had TB in the last 3 months, and they have not been diagnosed with AIDS yet, then there is a 2-fold increased risk of death (but not of AIDS).

### Independent effect of having a current ADC

The risk of death (all causes of death, excluding non-HIV causes) increases 2-fold if they currently have an ADC.

## Rate of occurrence of death from non-HIV causes

Rates from UK national mortality statistics are used.

### Increased risk of death for people with HIV

There is increasing evidence that people with HIV infection itself may have a raised risk of common clinical conditions such as non-AIDS cancers, renal and liver disease and cardiovascular diseases (30-35). Data from observational studies suggest that there is a modest increased risk of death for HIV-positive people with CD4 count greater than 500/mm3, compared to the general population, of the order of approximately 1.5 (36;37). Hence, we also assumed that there was a 1.5-fold increased rate of all non-HIV causes of death throughout life.

### Effect of smoking

Smokers experience 1.5-fold increased rate of non-HIV deaths.

Non-smokers experience 0.75-fold increased rate of non-HIV deaths (i.e. decreased risk of death).

This is consistent with a two-fold increase in all-cause mortality associated with smoking (1).

| Independent effect on rates |  | AIDS | CDC B  Symptoms | HIV-related  deaths | Liver death  (for those with  HBV or HCV) | Non-HIV deaths |
| --- | --- | --- | --- | --- | --- | --- |
|  |  | Dependent on most recent CD4 count (see 2.5.1) | Rate of AIDS  x 8 | Rate of AIDS  ÷ 4 | Rate of HIV -related deaths ÷ 6 | Rates from country  -specific national  mortality statistics x 1.5 |
|  |  |  |  |  |  |  |
| Effect of VL (log/copies) | < 3 | x 0.2 | x 0.2 | x 0.2 |  |  |
|  | 3 - 3.99 | x 0.3 | x 0.3 | x 0.3 |  |  |
|  | 4 - 4.49 | x 0.6 | x 0.6 | x 0.6 |  |  |
|  | 4.5 - 4.99 | x 0.9 | x 0.9 | x 0.9 |  |  |
|  | 5 - 5.49 | x 1.2 | x 1.2 | x 1.2 |  |  |
|  | ≥ 5.5 | x 1.6 | x 1.6 | x 1.6 |  |  |
|  |  |  |  |  |  |  |
| Effect of Age |  | x (age÷38)1.2 | x (age÷38)1.2 | x (age÷38)1.2 |  |  |
|  |  |  |  |  |  |  |
| Effect of PCP Prophylaxis |  | x 0.8 |  | x 0.8 |  |  |
|  |  |  |  |  |  |  |
| Effect of number of drugs in regimen | 1 drug | x 0.9 | x 0.9 | x 0.9 |  |  |
|  | 2 drugs | x 0.85 | x 0.85 | x 0.85 |  |  |
|  | 3 drugs | x 0.8 | x 0.8 | x 0.8 |  |  |
|  |  |  |  |  |  |  |
| Effect of smoking | Smoker |  |  |  |  | x 1.5 |
|  | Non-smoker |  |  |  |  | x 0.75 |

## Summary table of rates (per year)

## Model fits which are relevant to the natural history

### Incubation period to AIDS and death from seroconversion (no ART) Observed data from reference (38).

| Year from s/c | % with AIDS | | % died | |
| --- | --- | --- | --- | --- |
|  | Observed | Model | Observed | Model |
| 1 | 0.6 | **0.7** | 0.3 | **0.6** |
| 2 | 2.0 | **2.4** | 1.4 | **1.6** |
| 3 | 4.3 | **5.6** | 3.1 | **3.3** |
| 4 | 8.1 | **10.9** | 5.8 | **6.5** |
| 5 | 13.4 | **17.2** | 9.8 | **11.5** |
| 6 | 19.8 | **23.6** | 14.8 | **17.1** |
| 7 | 25.9 | **30.3** | 20.5 | **23.4** |
| 8 | 32.3 | **37.0** | 27.0 | **30.5** |
| 9 | 38.8 | **43.4** | 33.8 | **38.1** |
| 10 | 46.1 | **49.7** | 40.5 | **45.2** |
| 11 | 53.0 | **55.4** | 48.3 | **51.5** |
| 12 | 58.1 | **60.6** | 55.4 | **57.5** |
| 13 | 63.0 | **66.0** | 62.4 | **63.9** |

### Incubation period to CD4 <200, <350, <500 (no ART) Observed data from reference (39).

| Year from s/c | % CD4 < 200 | | % CD4 < 350 | | % CD4 < 500 | |
| --- | --- | --- | --- | --- | --- | --- |
|  | Observed | Model | Observed | Model | Observed | Model |
| 1 | 8.8 | **2.4** | 26.1 | **16.2** | 48.0 | **47.1** |
| 2 | 12.2 | **10.7** | 33.2 | **32.2** | 55.9 | **61.2** |
| 5 | 32.3 | **38.7** | 55.0 | **60.0** | 72.7 | **78.7** |

### Viral load set point and initial CD4 count (after primary infection) Observed data from reference (40).

|  | Observed | Model |
| --- | --- | --- |
| Median VL set point: | 4.5 | **4.0** |
| Median CD4: | 570 | **576** |

### Incubation period AIDS to death (pre-ART era) Observed data from reference (20).

|  | % died | |
| --- | --- | --- |
| Years from AIDS diagnosis | observed | Model |
| 1 | 40% | **39%** |
| 3 | 84% | **73%** |
| Median | 17 months | **18 months** |

### Association between viral load measured close to seroconversion (between 6-24 months) and risk of AIDS, adjusting for CD4 count and age. Observed data from reference (12).

|  | Adjusted Relative Hazard | |
| --- | --- | --- |
|  | Observed (95% confidence interval) | **Model** |
| Viral load (per 0.5 log higher) | 1.87 (1.58 – 2.20) | **2.15** |
| CD4 count (per 100 cells/mm3 lower) | 1.12 (1.02 – 1.24) | **1.14** |
| Age (per 10 years older) | 1.19 (0.96 – 1.47) | **1.28** |

### Risk of AIDS by CD4 count and viral load and age over 6 years (pre-HAART) Observed data from reference (17).

|  | Viral load | Observed | **Model** |
| --- | --- | --- | --- |
|  | < 1500 - (low n) |  |  |
|  | 1501- 7000 | 19 | **46** |
| CD4 < 350 | 7001- 20000 | 42 | **59** |
|  | 20001- 55000 | 73 | **79** |
|  | > 55000 | 92 | **94** |
|  | < 1500 - (low n) |  |  |
|  | 1501- 7000 | 22 | **18** |
| CD4 350-500 | 7001- 20000 | 40 | **37** |
|  | 20001- 55000 | 57 | **55** |
|  | > 55000 | 78 | **74** |
|  | < 1500 - (low n) | 5 | **5** |
|  | 1501- 7000 | 15 | **6** |
| CD4 > 500 | 7001- 20000 | 26 | **17** |
|  | 20001- 55000 | 48 | **28** |
|  | > 55000 | 67 | **60** |

* Viral load values used in MACS may need to be multiplied by ~ 2 to approximate to more commonly used Roche assay levels.

### Median CD4 count at diagnosis of AIDS and at death (pre-HAART era) Observed data from reference (19).

|  | AIDS | death |
| --- | --- | --- |
| Observed: | ~ 40 | ~ 0 |
| Model: | **36**  **IQR 10 - 107** | **5**  **IQR 1 - 28** |

# Effect of ART

## Adherence

An individual’s overall adherence to ART is summarised by the concept of ‘effective adherence’, which reflects the predicted adequacy of drug levels. Along with other factors, the ‘effective adherence’ determines the changes in CD4 cell count, viral load and risk of resistance mutation accumulation whilst a patient is on ART (as seen in section 3.3).

The ‘effective adherence’ depends on a number of components, some of which are fixed for each individual and some of which vary from period-to-period:

‘Effective adherence’ = Person’s underlying tendency to adhere
 + period-to-period variability in person’s underlying tendency to adhere

+ effect of calendar year

+ effect of receipt of NNRTI-based regimen

+ effect of experience of an occasional severe drop in adherence

The rationale for including these components and the values uses are described in the sections below.

### Underlying tendency to adhere and associated period-to-period variability

Each patient has an ‘*underlying tendency to adhere*’ to ART, which is a fixed value for each patient throughout their follow-up.

Each patient also has a ‘*within person period-to-period variability*’ to adhere. The magnitude of this variability is co-determined with the ‘underlying tendency to adhere’. Similar to the ‘underlying tendency to adhere’, the ‘within person period-to-period variability’ is also a fixed value for each patient throughout their follow-up. The values used for the base model are as shown below.

| Adherence | ‘Underlying tendency  to adhere’ | Probability of given level of ‘underlying tendency to adherence’ | ‘Within person  (period-to-period) variability’  in adherence  (standard deviation) |
| --- | --- | --- | --- |
| Low | 0.49 | 5% | 0.2 |
| Moderate | 0.8 | 10% | 0.2 |
| High­ | 0.9 | 65% | 0.06 |
| Very High | 0.95 | 20% | 0.05 |

A patient’s ‘underlying tendency to adhere’ is restricted to between 0 and 1 inclusive.

**Comment:** These estimates are based partially on observed adherence data (41-46), but also on adherence levels required to produce observed estimates of rates of resistance development and virologic failure (see model fit below) and also data on the proportion of patients at first virologic failure who have no resistance mutations present (47). It is clear from such data in more recent years that the great majority of patients who started ART with three or more drugs are sufficiently adherent that virologic failure rates are low (and so resistance accumulation is also likely to have been slow) (48;49).

### Effect of calendar year

The adherence at time *t* varies from period-to-period. The magnitude of deduction in adherence depends on the current calendar year in the following way:

| Calendar year of infection | Difference in  adherence |
| --- | --- |
| Before 1996 | -0.12 |
| 1996 | -0.10 |
| 1997 | -0.09 |
| 1998 | -0.08 |
| 1999 | -0.07 |
| 2000 | -0.06 |
| 2001 | -0.05 |
| 2002 | -0.04 |
| 2003 | -0.03 |
| 2004 | -0.02 |
| 2005 | -0.01 |
| 2006 | -0.00 |
| 2007 | +0.01 |
| 2008 | +0.02 |
| 2009 | +0.03 |
| 2010 | +0.04 |

Adherence is assumed to have improved over time as the understanding of the need for complete adherence and adherence support has evolved. This is supported by analyses showing improvements over calendar time in observed viral load levels on ART, even for a given specific regimen (48;49).

### Effect of receipt of NNRTI-based regimen

If a patient is on an NNRTI-based regimen (i.e. efavirenz), then the patient’s ‘effective adherence’ increases by 0.1. This effect is included in order to reflect the long half life of NNRTIs (50). This factor is the reason why we refer to the variable as ‘*effective* adherence’.

In addition, an individual’s ‘effective adherence’ is capped at 0.5 and will not be any lower, if they are on efavirenz and they have more than 0 active drugs (see section 4.3.7 for the concept of number of active drugs in the model). This has been put in so that people have a high risk of resistance with NNRTIs even if they have very low adherence.

### Effect of experience of an occasional severe drop in adherence

It is assumed that patients on ART are susceptible to occasional severe temporary drops in drug level (effective adherence level) at a rate of 0.02 events per year. On these occasions, the patient’s ‘effective adherence’ declines by 0.6. This leaves them susceptible to viral rebound (but with low risk of resistance, as the effective adherence drop is so profound it would put them in the lowest ‘effective adherence’ category).

This phenomenon is assumed to be 3 times more frequent among those on PI regimens. This latter assumption is what we consider the only plausible means (at least within our model framework) to explain why virologic failure occurring on boosted PI regimens often occurs in the absence of any resistance (51-53).

### Calculation of ‘effective adherence’

The ‘effective adherence’ is calculated for each person at each time point (every 3 months) whilst they are receiving ART.

Once all the components which affect ‘effective adherence’ (seen in sections 3.1.1-3.1.4) have been taken into account, the final value of ‘effective adherence’ is restricted to between 0 and 1 inclusive.

Example situation

The ‘effective adherence’ for a person receiving and taking ART in the year 2000, who has a high ‘underlying tendency to adhere’, and who is on an efavirenz-containing regimen currently, is:

0.9 [high ‘underlying tendency to adhere’]

+ Normal(0,0.06) [‘Within person (period-to-period) variability’]

+ (-0.06) [calendar year = 2000]

+ 0.05 [on efavirenz-containing regimen]

(capped at 0 if this value is < 0 and capped at 1 if this value is > 1).

## Use of antiretroviral therapy (ART)

### In the MSM Transmission model, the following antiretroviral drugs were considered:

| Drug class | Drug name |
| --- | --- |
| Nucleoside reverse transcriptase inhibitors (NRTIs) | zidovudine |
|  | didanosine (ddI) |
|  | lamivudine (3tc) |
|  | tenofovir |
| Non-nucleoside reverse transcriptase inhibitors (NNRTIs) | efavirenz |
| Protease Inhibitors (PIs) | indinavir |
|  | lopinavir/r |
|  | darunavir |
| Integrase Inhibitor | raltegravir |

Indinavir is assumed to be ritonavir-boosted after 2003.5.

**Comment:** These assumptions are based on references (54-58).

## Effect of ART

Potent ART regimens are known to reduce viral load, which in turn leads to recovery of CD4 cell counts (49;59;60). Changes in the viral load and CD4 counts are modelled separately when an individual is on ART (whereas in a situation in which the individual is ART-naïve, the CD4 changes are dependent on most recent viral load measurement – see section 2).

### Determination of viral load and CD4 count changes between *t-1* and *t*

The change in viral load between *t-1* and *t* depends only on the ‘effective adherence’, the number of active drugs (see section 4) and time on the current regimen. The change in CD4 count between *t-1* and *t* depends on the ‘effective adherence’, the number of active drugs, time on the current regimen, but also the relative propensity for CD4 rise for that individual. The way in these values are generated are detailed on the following pages.

Risk of development of resistance mutations is also dealt with concomitantly, using the same strata. However, these details are left out of here to make the document more manageable and are considered in section 4.

In the following sections, ‘starting current regimen’ means starting treatment for the first time as well as any treatment regimen following a treatment interruption.

**Comment:** Changes in viral load and CD4 count are based on observed data and observational studies (and to some extent randomized trials, although responses tend to be better in trial participants), and together with the modelling of development of resistance mutations, provide longer term estimates of virologic failure rates and CD4 count increases in ART. These changes documented here are broadly consistent with observed data (59-64).

#### Changes in viral load and CD4 count in first 3 months since starting current regimen.

The initial 3-month change in viral load is described as the mean change from the patient’s maximum viral load to that point (*vmax*) on the log scale. This is the mean of a normal distribution with standard deviation 0.2, from which the patient’s value/change is sampled.

The change in CD4 count is described as the mean change between periods *t-1* and *t*. This change is then multiplied by a factor which represents each individual’s underlying propensity for CD4 count rise whilst on ART (calculation of the factor is described in section 3.3.2.1). If the mean CD4 count change obtained from the table below is positive, then the mean value is subsequently multiplied by this factor.
However, if the CD4 count change in the table is a negative value (i.e. not a CD4 count rise), then it is not multiplied by this factor. Once the mean of the underlying CD4 count is obtained, to obtain the (underlying) CD4 count, variability (SD = 1.2) is added on the square root scale.

|  |  | Number of active drugs | | | | | | | | | | | | |
| --- | --- | --- | --- | --- | --- | --- | --- | --- | --- | --- | --- | --- | --- | --- |
|  | ‘Effective  adherence’ between *t-1* & *t* | 3 | 2.75 | 2.5 | 2.25 | 2 | 1.75 | 1.5 | 1.25 | 1 | 0.75 | 0.5 | 0.25 |  |
| Viral load (log change from *vmax*) | > 0.8 | -3 | -2.6 | -2.2 | -1.8 | -1.5 | -1.25 | -0.9 | -0.8 | -0.7 | -0.55 | -0.4 | -0.3 |  |
|  | > 0.5, < 0.8 | -2 | -1.6 | -1.2 | -1.1 | -0.9 | -0.8 | -0.6 | -0.5 | -0.4 | -0.25 | -0.1 | -0.05 |  |
|  | < 0.5 | -0.5 | -0.4 | -0.3 | -0.25 | -0.2 | -0.15 | 0 | 0.05 | 0.1 | 0.1 | 0.1 | 0.1 |  |
|  |  |  |  |  |  |  |  |  |  |  |  |  |  |  |
| CD4 count change (*t-1* to *t*) | > 0.8 | 70 | 45 | 40 | 35 | 30 | 25 | 20 | 17 | 13 | 10 | 5 | -2 |  |
|  | > 0.5, < 0.8 | 30 | 30 | 23 | 20 | 15 | 13 | 10 | 8 | 5 | 3 | 0 | -7 |  |
|  | < 0.5 | 5 | 4 | 3 | 2 | 1 | -1 | -3 | -6 | -10 | -11 | -12 | -13 |  |

#### Summary of viral load between 3-6 months since starting current regimen and after 6 months if viral load at *t-1* > 4 log copies/ml.

This table applies to patients for whom it has been between 3 and 6 months since starting their current regimen, as well as patients who have been on their current regimen for more than 6 months but who have a viral load > 4 log copies/ml (e.g. due to previous poor adherence).

The change in viral load is described as the mean change from the patient’s maximum viral load to that point (*vmax*) on the log scale. Otherwise, if the number in the table is underlined, it is the mean absolute value. This is the mean of a normal distribution with standard deviation 0.2, from which the patient’s value/change is sampled.

|  |  | Number of active drugs | | | | | | | | | | | |
| --- | --- | --- | --- | --- | --- | --- | --- | --- | --- | --- | --- | --- | --- |
| ‘Effective adherence’ between *t-2* & *t-1* | ‘Effective adherence’ between *t-1* & *t* | 3 | 2.75 | 2.5 | 2.25 | 2 | 1.75 | 1.5 | 1.25 | 1 | 0.75 | 0.5 | 0.25 |
| > 0.8 | > 0.8 | 0.5 | 0.8 | 1.2 | 1.4 | 2.0 | 2.7 | -1.7 | -1.15 | -0.9 | -0.75 | -0.6 | -0.4 |
| > 0.5, < 0.8 | > 0.8 | 1.2 | 1.2 | 1.2 | 1.4 | -2.0 | -1.6 | -1.2 | -1.05 | -0.9 | -0.7 | -0.5 | -0.35 |
| < 0.5 | > 0.8 | 1.2 | 1.2 | 1.2 | 1.4 | -2.0 | -1.6 | -1.2 | -1.0 | -0.9 | -0.7 | -0.5 | -0.2 |
|  |  |  |  |  |  |  |  |  |  |  |  |  |  |
| > 0.8 | > 0.5, < 0.8 | 1.2 | 1.6 | 1.8 | 2.2 | 2.4 | -2.4 | -1.5 | -0.9 | -0.7 | -0.55 | -0.4 | -0.3 |
| > 0.5, < 0.8 | > 0.5, < 0.8 | 2.5 | 2.5 | 2.5 | 2.5 | -1.2 | -1.1 | -0.8 | -0.65 | -0.5 | -0.35 | -0.2 | -0.05 |
| < 0.5 | > 0.5, < 0.8 | -2.0 | -1.8 | -1.5 | -1.35 | -1.2 | -1.1 | -0.8 | -0.65 | -0.5 | -0.2 | -0.2 | -0.05 |
|  |  |  |  |  |  |  |  |  |  |  |  |  |  |
| > 0.8 | < 0.5 | -0.5 | -0.4 | -0.3 | -0.25 | -0.2 | -0.15 | -0.10 | -0.05 | +0 | +0 | +0 | +0 |
| > 0.5, < 0.8 | < 0.5 | -0.5 | -0.4 | -0.3 | -0.25 | -0.2 | -0.15 | -0.10 | -0.05 | +0 | +0 | +0 | +0 |
| < 0.5 | < 0.5 | -0.5 | -0.4 | -0.3 | -0.25 | -0.2 | -0.15 | -0.10 | -0.05 | +0 | +0 | +0 | +0 |

#### Summary of CD4 count change (mean change between *t-1* and *t*) between 3-6 months since starting current regimen and after 6 months if viral load at *t-1* > 4 log copies/ml.

This table applies to patients for whom it has been between 3 and 6 months since starting their current regimen, as well as patients who have been on their current regimen for more than 6 months but who have a viral load > 4 log/copies/ml (e.g. due to previous poor adherence).

The change in CD4 count is described as the mean change between periods *t-1* and *t*. This change is then multiplied by a factor which represents each individual’s underlying propensity for CD4 count rise whilst on ART (calculation of the factor is described in section 3.3.2.1). If the mean CD4 count change obtained from the table below is positive, then the mean value is subsequently multiplied by this factor.
However, if the CD4 count change in the table is a negative value (i.e. not a CD4 count rise), then it is not multiplied by this factor. Once the mean of the underlying CD4 count is obtained, to obtain the (underlying) CD4 count, variability (SD = 1.2) is added on the square root scale.

|  |  | Number of active drugs | | | | | | | | | | | |
| --- | --- | --- | --- | --- | --- | --- | --- | --- | --- | --- | --- | --- | --- |
| ‘Effective adherence’ between *t-2* & *t-1* | ‘Effective adherence’ between *t-1* & *t* | 3 | 2.75 | 2.5 | 2.25 | 2 | 1.75 | 1.5 | 1.25 | 1 | 0.75 | 0.5 | 0.25 |
| > 0.8 | > 0.8 | +30 | +28 | +25 | +23 | +21 | +19 | +3 | -5 | -9 | -10.5 | -12 | -14 |
| > 0.5, < 0.8 | > 0.8 | +30 | +28 | +25 | +23 | +7.5 | +1.5 | -4.5 | -7 | -9 | -11 | -13 | -14.5 |
| < 0.5 | > 0.8 | +30 | +28 | +25 | +23 | +7.5 | +1.5 | -4.5 | -7.5 | -9 | -11 | -13 | -16 |
|  |  |  |  |  |  |  |  |  |  |  |  |  |  |
| > 0.8 | > 0.5, < 0.8 | +15 | +13 | +10 | +8 | +7 | +4 | +0 | -9 | -11 | -12.5 | -14 | -15 |
| > 0.5, < 0.8 | > 0.5, < 0.8 | +15 | +13 | +10 | +8 | -4.5 | -6 | -10 | -11.5 | -13 | -14.5 | -16 | -17.5 |
| < 0.5 | > 0.5, < 0.8 | +7.5 | +4.5 | +0 | -2 | -4.5 | -6 | -10 | -11.5 | -13 | -16 | -16 | -17.5 |
|  |  |  |  |  |  |  |  |  |  |  |  |  |  |
| > 0.8 | < 0.5 | -13 | -14 | -15 | -15.5 | -16 | -16.5 | -17 | -17.5 | -18 | -18 | -18 | -18 |
| > 0.5, < 0.8 | < 0.5 | -13 | -14 | -15 | -15.5 | -16 | -16.5 | -17 | -17.5 | -18 | -18 | -18 | -18 |
| < 0.5 | < 0.5 | -13 | -14 | -15 | -15.5 | -16 | -16.5 | -17 | -17.5 | -18 | -18 | -18 | -18 |

#### Summary of viral load (mean change from viral load max), CD4 count change (mean change between *t-1* and *t*), after 6 months, where viral load at *t-1* < 4 log copies/ml.

The change in viral load and CD4 count is as described previously (sections 3.3.1.2 and 3.3.1.3).

|  |  | Number of active drugs | | | | | | | | | | | |
| --- | --- | --- | --- | --- | --- | --- | --- | --- | --- | --- | --- | --- | --- |
|  | ‘Effective  adherence’ between *t-1* & *t* | 3 | 2.75 | 2.5 | 2.25 | 2 | 1.75 | 1.5 | 1.25 | 1 | 0.75 | 0.5 | 0.25 |
| Viral load (log change from *vmax*) | > 0.8 | 0.5 | 0.9 | 1.2 | 1.6 | -2.5 | -2.0 | -1.4 | -1.15 | -0.9 | -0.75 | -0.6 | -0.3 |
|  | > 0.5, < 0.8 | 1.2 | 1.2 | 1.2 | 1.4 | -1.2 | -1.0 | -0.7 | -0.6 | -0.5 | -0.4 | -0.3 | -0.1 |
|  | < 0.5 | -0.5 | -0.4 | -0.3 | -0.25 | -0.2 | -0.2 | -0.1 | -0.1 | -0.1 | -0.1 | -0.1 | 0 |
|  |  |  |  |  |  |  |  |  |  |  |  |  |  |
| CD4 count change (*t-1* to *t*) | > 0.8 | +30 | +28 | +25 | +23 | +21 | +19 | +3 | -5 | -9 | -10.5 | -12 | -12 |
|  | > 0.5, < 0.8 | +15 | +13 | +10 | +8 | -4.5 | -7.5 | -10 | -12 | -13 | -14 | -15 | -15 |
|  | < 0.5 | -13 | -14 | -15 | -15.5 | -16 | -16.5 | -17 | -17 | -18 | -17 | -17 | -17 |

#### Changes in viral load and CD4 count if the number of active drugs in current regimen = 0.

For 0 active drugs, these are the changes regardless of time from start of regimen.

|  |  | Number of active drugs |
| --- | --- | --- |
|  | ‘Effective  adherence’ between *t-1* & *t* | 0 |
| Viral load (log change from *vmax*) | > 0.8 | -0.3 |
|  | > 0.5, < 0.8 | -0.1 |
|  | < 0.5 | 0 |
|  |  |  |
| CD4 count change (*t-1* to *t*) | > 0.8 | -15 |
|  | > 0.5, < 0.8 | -17 |
|  | < 0.5 | -18 |

### Other effects of ART on CD4 count

#### Relative propensity for CD4 count rise

Patients are assumed to vary in their underlying propensity for CD4 count rise whilst on ART. Each person is given a value for their propensity, ‘*patient_CD4_rise*’, which is sampled from exp(Normal(0,0.5)), i.e. log-normal distribution with mean 0 and standard deviation 0.5.

Thus, ‘patient_CD4_rise’is distributed approximately as follows:

‘patient_CD4_rise’ is a fixed value that remains constant for the individual over time and is the factor by which the CD4 count change is multiplied by in sections 3.3.1.1 and 3.3.1.3.

If a patient has been on their current regimen for longer than 2 years, their underlying propensity for CD4 count rise reduces 4-fold, to reflect the fact that the rate of CD4 count increase decreases over time (65;66).

#### CD4 decline with failing NNRTI-regimen

When a patient is on an NNRTI-regimen (i.e. efavirenz) but not on a PI (i.e. lopinavir), and they are failing the regimen, then the CD4 depletion is greater (by -10 cells/mm3) in any given 3-month period. Failure of the regimen is defined in this situation by a number of active drugs (concept explained under 4.3.7) being less than or equal to 2.

#### Variability in CD4 count whilst on ART

When a person is ART-naïve, the CD4 count change from period *t-1* to *t* (i.e. in a 3 month period) is given by sampling from a normal distribution with mean dependent on viral load at *t-1*, and standard deviation 1.2 on the square root scale (see section 2.3).

When a person is on ART, the same magnitude of variability (i.e. standard deviation of 1.2 on the square root scale) as for when the person is ART-naïve, is given to the CD4 count change. (This variability has already been described above in sections 3.3.1.1 and 3.3.1.3)

**Comment:** Note that this is not the same as the variability given to *measured* CD4 count (which has a standard deviation of 2.0 on the square root scale, as found in section 2), which applies to people both on and off ART.

#### Maximum CD4 count

The maximum potentially attainable CD4 count in people on ART is distributed as follows (i.e. Normal(800,150)):

**Comment:** This estimate is based on observed CD4 counts in HIV-seronegative people. See references (67;68).

After all the effects of ART (as seen above) have been taken into account to calculate the CD4 count, if the CD4 count is greater than the maximum CD4 count assigned for that person, then a further expression is used to calculate the resulting CD4 count at time *t*:

Final CD4 at time *t* = (Maximum CD4 count) + Normal(0,50)

#### Minimum CD4 count

As mentioned in section 1, the minimum CD4 count and minimum measured CD4 count are both defined to be 0.

#### Calculation of measured CD4 count (whilst on ART)

The measured CD4 count is calculated for each person at every time point (3 months). The example below applies only to individuals who are taking ART.

Once all the components which affect measured CD4 count (seen in sections 3.3.1-3.3.2.2) have been taken into account, the final value of measured CD4 count is restricted to between 0 and the maximum CD4 count as calculated in section 3.3.2.4.

Example situation

The measured CD4 count for a person at time *t*, given that their actual CD4 count and viral load at *t-1* was 350 cells/mm3 and 4.1 log copies/ml respectively, and they have been on their current regimen for over 6 months, where the number of active drugs in their regimen totals 2.50, and given that their ‘effective adherence’ has been consistently over 0.8, and their underlying propensity for CD4 count rise (patient_cd4_rise) = 1.1, is:

( √ (√ (350 + (25 x 1.1))+Normal(0,1.2) )2 + Normal(0,2.0) )2

where +25 is the CD4 change obtained from section 3.1.3, Normal(0,1.2) is the variability given to the change in CD4 whilst on ART, and Normal(0,2.0) is the variability given for *measured* CD4 count.

(capped at 0 if this value is < 0 and capped at (Maximum CD4 count) + Normal(0,50) if this value is > assigned maximum CD4 for that person).

## Model fits which are relevant to the effect of ART

### Mean changes from baseline in CD4 cell count and viral load whilst on zidovudine mono-therapy (patients with no previous treatment) Observed data from references (69;70)

### Mean changes from baseline in CD4 cell count and viral load whilst on zidovudine/3TC dual-therapy (patients with no previous treatment) Observed data from reference (69)

### 3 year percent risk of AIDS after start of ART by baseline CD4 / viral load (age < 50, non-IDU, AIDS-free) Observed data from reference (71).

| Baseline viral load | Baseline CD4 count | Observed | **Model** |
| --- | --- | --- | --- |
|  | < 50 | 16% | **23%** |
|  | 50-99 | 12% | **15%** |
| < 100,000 | 100-199 | 9% | **8%** |
|  | 200-349 | 5% | **6%** |
|  | > 350 | 3% | **2%** |
|  | < 50 | 20% | **18%** |
|  | 50-99 | 16% | **16%** |
| > 100,000 | 100-199 | 12% | **4%** |
|  | 200-349 | 6% | **4%** |
|  | > 350 | 4% | **0%** |

### Effect of HAART vs no therapy on risk of AIDS and death Observed data from reference (72).

Simulated trial with 5 years follow-up.

Relative hazard of AIDS, (HAART vs no therapy)

| Observed | **Model** |
| --- | --- |
| 0.1 | **0.16** |

### % with virologic failure (viral load > 500 copies/mL, whilst on ART) by time from start of HAART (patients starting with PI/r or NNRTI regimen) Observed data from reference (73).

| Years from start of HAART | Observed | **Model** |
| --- | --- | --- |
| 1 | 7% | **10%** |
| 2 | 13% | **16%** |
| 3 | 17% | **19%** |
| 4 | 20% | **23%** |
| 5 | 22% | **26%** |
| 6 | 24% | **28%** |
| 7 | 27% | **30%** |
| 8 | 29% | **32%** |

Observed data may be overestimates due to some unrecognised stopping of ART.

### Rate of viral rebound in people on 1st line HAART and with viral load < 50 copies/mL Observed data from reference (74).

|  | Rate per 100 person -years |
| --- | --- |
| Observed | 3-6 |
| **Model** | **5.5** |

### Median CD4 count change (cells/mm3) at 3 years from start of HAART Observed data from reference (63).

| Observed | **Model** |
| --- | --- |
| 273 | **274** |

### Percent with triple class virologic failure by years from start of HAART (patients naïve before HAART) Observed data from reference (75).

Modelled estimates based on ART start years 1998-2008 inclusive.

| Years from start of HAART | Observed | **Model** |
| --- | --- | --- |
| 5 | 3.4% | **6.8%** |
| 9 | 8.6% | **12.6%** |

# HIV drug resistance

## Resistance mutations; introduction

### Resistance mutations included in the model

The resistance mutations considered in the model are as follows. We do not specify the mutated amino acid for each position; it is assumed that for a given codon position, the mutations considered are those that confer resistance (e.g. for M184 this is I or V). The exception to this are the mutations at codon 50 of protease inhibitors – we may need to review other mutations to see if they need to be split, but there is a cost to adding any new variables.

The choice of mutations to include reflects a balance between the desire to capture important specific effects and the need to limit the complexity of the model and the number of variables simulated. The IAS-USA resistance guidelines provided the basis for choice of mutations (76).

| Drug class | Resistance mutations | Notes |
| --- | --- | --- |
| NRTI | M184 |  |
|  | TAMS | TAMS (thymidine analog mutations) are modelled such that it is the number of TAMS which affect the drug activity, rather than the specific mutations itself |
|  | K65 |  |
|  | L74 |  |
|  | Q151 |  |
|  |  |  |
| NNRTI | nn | A specific resistance mutation (or mutations) which confers resistance to an NNRTI drug |
|  |  |  |
| PI | V32 |  |
|  | M46 |  |
|  | I47 |  |
|  | I50V |  |
|  | I54 |  |
|  | L76 |  |
|  | V82 |  |
|  | I84 |  |
|  |  |  |
| Integrase Inhibitor | CCR5m | A specific resistance mutation to a CCR5 antagonist drug  (this is separate from presence of X4 virus, which is modelled separately) |

**Comment:** Note that the possibility of mutations to anticipated drugs is accounted for. This is necessarily crude (as the new drugs that will be licensed and their resistance profiles are as yet uncertain) but conveys the fact that new drugs are under development for which the virus will have to develop new mutations to evade. However, it is clear that we are now in a position to be more specific about mutations to some new drugs, such as raltegravir, and therefore update the model.

### Types of variables used to model resistance mutations

Resistance mutations can be present in majority or minority virus and this is also reflected in the model. In the following sections, *resistancemutation* can represent any of the mutations as listed in the table in section 4.1:

c_*resistancemutation* = 1 if mutation is present in majority virus

= 0 if not

e_*resistancemutation* = 1 if virus with mutation is present at all

= 0 if not

Once e_*resistancemutation* takes the value 1, it can never revert to 0.

Note: Unlike all other resistance mutations, M184 is assumed not to persist in majority virus after HIV infection; although like all other mutations, it does persist as minority virus.

## ART-naïve patients

### Transmitted drug resistance

There is a possibility of resistance mutations being present in the acquired virus (transmitted drug resistance) at the point of infection.

The viral load group of the man who infected the subject is known, as indicated above (for infection from a short term partner the viral load group of the 6 groups defined in section 1.2.5 is known, while if infected by a longer term partner the viral load is known to be either < 2.7, > 2.7 but not primary infection, or primary infection, as described in section 1.2.6). For a subject infected by a person in viral load group v the probability of a resistance mutation being present in the infected person is given by

Σv,r=1 L1(t-1) / Σv L1(t-1)

where Σv, r=1 is the sum over all HIV-infected subjects in viral load group v for whom a resistance mutation is present in majority virus and Σ is the sum over all HIV-infected subjects in viral load group v. Again, realization of whether the subject is infected by a person with at least one resistance mutation in majority virus is determined by sampling from Uniform(0,1).

For subjects infected from a source partner with a resistance mutation, the probability that a specific mutation, m, is present in the source is given by

Σr=1,m=1 L1(*t-1*) / Σr=1 L1(*t-1*)

where Σr=1,m=1 is the sum over all HIV-infected subjects with mutation m present in majority virus and Σr=1 is the sum over all HIV-infected subjects with at least one resistance mutation in majority virus.

If a given resistance mutation, m, is present in the source partner, the probability that the mutation is both transmitted and survives in the subject (i.e. that its presence will affect future response to drugs for which the mutation confers reduced sensitivity) is mutation specific, as shown in the table below:

| Resistance mutation | Probability the mutation is both transmitted and survives in the subject |
| --- | --- |
| M184 | 0.05 |
| K65 | 0.50 |
| L74 | 0.50 |
| Q151 | 0.50 |
| Thymidine analogue mutations (TAMS) | 0.50 |
| NNRTI mutation | 0.50 |
| PI mutations | 0.50 |

**Comments:** Probabilities based on evidence from studies comparing distribution of resistance mutations between treated and antiretroviral naïve populations; e.g. refs (77;78).

## Patients on ART

### Abbreviations of antiretroviral drugs used in the model

NRTI:

ZDV/zidovudine, DDI/didanosine, 3TC/lamivudine, TEN/tenofovir

NNRTI:

EFA/efavirenz,

PI:

IND/indinavir, LPR/lopinavir, DAR/darunavir

Integrase Inhibitor:

RAL/raltegravir

### Determination of acquisition of new resistance mutations between *t-1* and *t*: Stage 1

For people on ART there is a certain risk of development of resistance mutations, which is determined by the number of active drugs in the regimen, viral load, the effective adherence (see section 3) and the time since starting (or re-starting after interruption) the current period of continuous therapy.

The probability of acquiring new resistance mutations in a given 3 month period in people on ART (i.e. between *t-1* and *t*) is determined in a series of stages. The first stage is to determine the ‘*new mutation factor’*. The magnitude of this factor reflects the risk of resistance mutations emerging, but it cannot in itself be interpreted as the risk of resistance emerging. The process for generating these values is described on the following pages.

The changes in viral load and CD4 count are also dealt with concomitantly, using the same strata. See section 3.3.1 for details.

#### ‘New mutation factor’ in first 3 months since starting current period of continuous therapy

The ‘new mutation factor’ is given in the table below.

|  | Number of active drugs | | | | | | | | | | | |
| --- | --- | --- | --- | --- | --- | --- | --- | --- | --- | --- | --- | --- |
| ‘Effective  adherence’ between *t-1* & *t* | 3 | 2.75 | 2.5 | 2.25 | 2 | 1.75 | 1.5 | 1.25 | 1 | 0.75 | 0.5 | 0.25 |
| > 0.8 | 0.002 | 0.01 | 0.03 | 0.05 | 0.1 | 0.15 | 0.2 | 0.3 | 0.4 | 0.45 | 0.5 | 0.5 |
| > 0.5, < 0.8 | 0.15 | 0.15 | 0.2 | 0.25 | 0.3 | 0.3 | 0.3 | 0.35 | 0.4 | 0.45 | 0.5 | 0.5 |
| < 0.5 | 0.05 | 0.05 | 0.05 | 0.05 | 0.05 | 0.05 | 0.05 | 0.05 | 0.05 | 0.05 | 0.05 | 0.05 |

#### Summary of ‘new mutation factor’ between 3-6 months since starting current period of continuous therapy and after 6 months if viral load at *t-1* > 4 log copies/ml

This table applies to patients for whom it has been between 3 and 6 months since starting their current period of continuous therapy, as well as for patients whom it has been more than 6 months since their current period of continuous therapy but who have a high viral load (e.g. due to previous poor adherence). The numbers given in the table below correspond to the ‘new mutation factor’.

|  |  | Number of active drugs | | | | | | | | | | | |
| --- | --- | --- | --- | --- | --- | --- | --- | --- | --- | --- | --- | --- | --- |
| ‘Effective adherence’ between *t-2* & *t-1* | ‘Effective adherence’ between *t-1* & *t* | 3 | 2.75 | 2.5 | 2.25 | 2 | 1.75 | 1.5 | 1.25 | 1 | 0.75 | 0.5 | 0.25 |
| > 0.8 | > 0.8 | 0.002 | 0.01 | 0.03 | 0.05 | 0.05 | 0.1 | 0.2 | 0.3 | 0.4 | 0.45 | 0.5 | 0.5 |
| > 0.5, < 0.8 | > 0.8 | 0.002 | 0.01 | 0.03 | 0.05 | 0.05 | 0.1 | 0.2 | 0.3 | 0.4 | 0.45 | 0.5 | 0.5 |
| < 0.5 | > 0.8 | 0.05 | 0.05 | 0.03 | 0.05 | 0.05 | 0.1 | 0.2 | 0.3 | 0.4 | 0.45 | 0.5 | 0.25 |
|  |  |  |  |  |  |  |  |  |  |  |  |  |  |
| > 0.8 | > 0.5, < 0.8 | 0.10 | 0.15 | 0.2 | 0.2 | 0.3 | 0.3 | 0.3 | 0.35 | 0.4 | 0.45 | 0.5 | 0.5 |
| > 0.5, < 0.8 | > 0.5, < 0.8 | 0.10 | 0.15 | 0.2 | 0.2 | 0.3 | 0.3 | 0.3 | 0.35 | 0.4 | 0.45 | 0.5 | 0.5 |
| < 0.5 | > 0.5, < 0.8 | 0.10 | 0.15 | 0.2 | 0.2 | 0.3 | 0.3 | 0.3 | 0.35 | 0.4 | 0.45 | 0.5 | 0.25 |
|  |  |  |  |  |  |  |  |  |  |  |  |  |  |
| > 0.8 | < 0.5 | 0.05 | 0.05 | 0.05 | 0.05 | 0.05 | 0.05 | 0.05 | 0.05 | 0.05 | 0.05 | 0.05 | 0.05 |
| > 0.5, < 0.8 | < 0.5 | 0.05 | 0.05 | 0.05 | 0.05 | 0.05 | 0.05 | 0.05 | 0.05 | 0.05 | 0.05 | 0.05 | 0.05 |
| < 0.5 | < 0.5 | 0.05 | 0.05 | 0.05 | 0.05 | 0.05 | 0.05 | 0.05 | 0.05 | 0.05 | 0.05 | 0.05 | 0.05 |

#### ‘New mutation factor’ after 6 months since starting current period of continuous therapy, where viral load at *t-1* < 4 log copies/ml.

The ‘new mutation factor’ is given in the table below.

|  | Number of active drugs | | | | | | | | | | | |
| --- | --- | --- | --- | --- | --- | --- | --- | --- | --- | --- | --- | --- |
| ‘Effective  adherence’ between *t-1* & *t* | 3 | 2.75 | 2.5 | 2.25 | 2 | 1.75 | 1.5 | 1.25 | 1 | 0.75 | 0.5 | 0.25 |
| > 0.8 | 0.002 | 0.01 | 0.03 | 0.08 | 0.1 | 0.15 | 0.2 | 0.3 | 0.4 | 0.45 | 0.5 | 0.5 |
| > 0.5, < 0.8 | 0.15 | 0.18 | 0.2 | 0.25 | 0.3 | 0.3 | 0.3 | 0.35 | 0.4 | 0.45 | 0.5 | 0.5 |
| < 0.5 | 0.05 | 0.05 | 0.05 | 0.05 | 0.05 | 0.05 | 0.05 | 0.05 | 0.05 | 0.05 | 0.05 | 0.05 |

#### ‘New mutation factor’ if the number of active drugs in current regimen = 0.

For 0 active drugs, these numbers below are the ‘new mutation factor’ regardless of time from start of current period of continuous therapy.

|  | Number of active drugs |
| --- | --- |
| ‘Effective  adherence’ between *t-1* & *t* | 0 |
| > 0.8 | 0.5 |
| > 0.5, < 0.8 | 0.5 |
| < 0.5 | 0.05 |

**Comment:** The ‘new mutation factor’ values under this section 4.3.2.4 probably need to be changed to 0, because when there are no active drugs, new resistance mutations should not in theory be selected for when they arise.

### Determination of acquisition of new resistance mutations between *t-1* and *t*:Stage 2

The second stage is for the ‘new mutation factor’ to be multiplied by the log viral load (mean of log viral load at *t-1* and *t*).

This is then converted to a number that can be used as a probability, by truncating at 1.

### Determination of acquisition of new resistance mutations between *t-1* and *t*:Stage 3

The value determined from Stage 2 (section 4.3.3) is used as a probability to determine whether the third stage is entered. If this chance arises in a given 3 month period, which is determined by sampling from the binomial distribution, then the following criteria operate:

#### NRTIs

| Resistance mutation | Probability of arising | Conditions |
| --- | --- | --- |
| M184 | 80% | if (on 3TC) |
| # TAMS increases by 1 | 20% | if (on ZDV) and (not on 3TC) |
|  | 12% | if (on ZDV) and (on 3TC) |
| # TAMS increases by 2 | 1% | if (on ZDV) and (not on 3TC) |
|  | 1% | if (on ZDV) and (on 3TC) |
| K65 | 1% | if (on TEN or DDI) and (on ZDV) |
|  | 4% | If (on TEN or DDI) and (not on ZDV) |
| L74 | 1% | if (on DDI) |
| Q151 | 2% | if (on DDI or ZDV) |

**Comment:** there are currently only 6 TAMS (according to IAS-USA mutations list, November 2011 update) (76).

#### NNRTIs

| Resistance mutation | Probability of arising | Conditions |
| --- | --- | --- |
| nn | 80% | if (on EFA) |

#### PIs

We assume a different probability of resistance mutation accumulation depending on whether the PI would be boosted or not (which for simplicity, we assume it depends entirely on the current calendar year).

| Resistance mutation | Probability of arising | Conditions |
| --- | --- | --- |
| V32 | 4% | if on LPR |
| M46 | 12% | If (on IND) and (year of infection < July 2000) |
|  | 4% | If (on IND) and (year of infection ≥ July 2000) |
| I47 | 4% | If on LPR |
|  | 4% | If on DAR |
| I54 | 4% | If on DAR |
| L76 | 4% | If on DAR |
| V82 | 12% | If (on IND) and (year of infection < July 2000) |
|  | 4% | If (on IND) and (year of infection ≥ July 2000) |
|  | 4% | If on LPR |
| I84 | 12% | If (on IND) and (year of infection < July 2000) |
|  | 4% | If (on IND) and (year of infection ≥ July 2000) |
|  | 4% | If on DAR |

#### Other classes

| Resistance mutation | Probability of arising | Conditions |
| --- | --- | --- |
| IIm | 80% | If on RAL |

**Comment:** Values of the ‘new mutation factor’ parameter have been chosen in conjunction with the translation of presence of mutations into reduced drug activity to provide estimates of accumulation of specific classes of resistance mutation broadly consistent with those observed in clinical practice (79;80). They reflect a greater propensity for some mutations to arise than others. This probability relates to the ability of the virus to replicate without the mutations (e.g. probably very low in the presence of 3TC for virus without M184) as well as the replicative capacity of virus with the mutations in the presence of treatment. Over time as more data accumulate, it may be possible improve these estimates of rates of accumulation of specific mutations.

### Determination of acquisition of new resistance mutations between *t-1* and *t*: Example calculation

Example

The probability of acquiring a new resistance mutation for a person who has been on their current treatment regimen for between 3-6 months, where their viral load at *t-1* and *t* were 4.5 and 4.7 respectively, who has consistently had an ‘effective adherence’ of over 0.8 and who has 2.75 active drugs in their current regimen, is:

0.01 [‘new mutation factor’ as seen in table 3.2.2]

x (4.5 + 4.7)/2 [mean of log viral load at *t-1* and *t*]

(capped at 1 if this value is > 1).

The resulting number is the probability used in Stage 3 (section 4.3.4) to decide whether there is a chance of any resistance mutations developing in the next 3 month period.

### Determination of level of activity for each drug

In what follows, note that, for example:

r_ZDV denotes the level of resistance to ZDV and can take the value 0, 0.25, 0.5, 0.75 or 1.

r_ZDV = 0 if there is no resistance to ZDV at time *t*

= 0.25, 0.5 or 0.75 if there is partial resistance to ZDV at time *t*

= 1 if there is complete resistance to ZDV at time *t*

#### NRTI - 3TC

| r_3TC | Mutations | Other conditions |
| --- | --- | --- |
| 0.75 | M184 | - |

The effect of TAMS is the same regardless of presence of r_3TC. This interaction is factored in earlier, at the level of reduced TAM accumulation when on 3TC.

#### NRTI - ZDV

| r_ZDV | Mutations | Other conditions |
| --- | --- | --- |
| 0.5 | 1 ≤ # of TAMS < 3 | Not on 3TC |
| 0.75 | 3 ≤ # of TAMS < 5 | Not on 3TC |
| 1 | # of TAMS ≥ 5 | Not on 3TC |
| 0.25 | 1 ≤ # of TAMS < 3, M184 | On 3TC |
| 0.5 | 3 ≤ # of TAMS < 5, M184 | On 3TC |
| 0.75 | # of TAMS ≥ 5, M184 | On 3TC |
| 0.5 | 1 ≤ # of TAMS < 3 | (On 3TC) and M184 mutation not present |
| 0.75 | 3 ≤ # of TAMS < 5 | (On 3TC) and M184 mutation not present |
| 0.75 | # of TAMS ≥ 5 | (On 3TC) and M184 mutation not present |
| 0.75 | Q151 | - |

#### NRTI - Tenofovir

| r_TEN | Mutations | Other conditions |
| --- | --- | --- |
| 0.5 | 2 ≤ # of TAMS ≤ 3 | (Not on 3TC)  or ((on 3TC) and M184 mutation not present)  and K65 mutation not present |
| 0.75 | # of TAMS ≥ 4 | (Not on 3TC)  or ((on 3TC) and M184 mutation not present)  and K65 mutation not present |
| 0.5 | # of TAMS ≥ 2, M184 | (on 3TC) and K65 mutation not present |
| 0.5 | K65 | - |

#### NRTI - DDI

| r_DDI | Mutations | Other conditions |
| --- | --- | --- |
| 0.75 | At least 1 mutation of (L74, K65) | - |
| 0.5 | # of TAMS ≥ 3 | - |
| 0.75 | Q151 | - |

#### NNRTIs

| r_EFA | Mutations | | Other conditions | |
| --- | --- | --- | --- | --- |
| 1 | | nn | | - |

#### PI - Indinavir

Distinguish by pre- and post- boosted.

| r_IND | Mutations | Other conditions |
| --- | --- | --- |
| 0.75 | At least 1 mutation of (M46, V82, I84) | Year of infection < July 2003 |
| 1 | M46, V82, I84 | Year of infection < July 2003 |
| 0.5 | 1 or 2 mutations of (M46, V82, I84) | Year of infection ≥ July 2003 |
| 0.75 | M46, V82, I84 | Year of infection ≥ July 2003 |

#### PI – Lopinavir

| r_LPR | Mutations | Other conditions |
| --- | --- | --- |
| 0.25 | 1 mutation of (V32, I47, L76, V82) | - |
| 0.5 | 2 mutations of (V32, I47, L76, V82) | - |
| 0.75 | V32, I47, L76, V82 | - |
| Max(r_LPR, 0.5) | M46, V82, I84, L90 | - |

#### PI – Darunavir

| r_DAR | Mutations | Other conditions |
| --- | --- | --- |
| 0.25 | 2 mutations of (I47, I50V, I54, L76, I84) | - |
| 0.5 | 3 mutations of (I47, I50V, I54, L76, I84) | - |
| 0.75 | At least 4 mutations of (I47, I50V, I54, L76, I84) | - |

#### II - Raltegravir

| r_RAL | Mutations | Other conditions |
| --- | --- | --- |
| 1 | IIm | - |

**Comment:** These rules approximately follow the interpretation systems for conversion of mutations present on genotypic resistance test into a predicted level of drug activity (or, equivalently, of resistance). Currently interpretation systems differ in their prediction of activity for some drugs. Over time as more data accumulate and interpretation systems converge it may be possible to refine these rules.

### Number of active drugs in the regimen

#### Calculation of the number of active drugs in the regimen

The activity level of a drug is given by

activity = 1 - level of resistance to the drug.

The number of active drugs in the regimen at time *t* is the sum of the activity level of each drug in the current regimen.

**Comment:** An implicit assumption here is that every drug is equally potent because virologic efficacy depends only on the number of active drugs and not which specific drugs are active. In reality, drugs differ in potency but to our knowledge, no reliable estimates are available to use (although further refinements of the model may use early phase data on the short-term (e.g. two weeks) effect of drugs on viral load when used as mono-therapy, as a measure of efficacy).

#### Total number of drugs, *nod*

The total number of drugs the patient is on at time *t*, is simply the sum of the number of antiretroviral drugs they are taking at that time.

In terms of ritonavir-boosted protease inhibitors, we do not count the ritonavir as a separate drug in this case.

#### Total resistance to ART, *nres*

The total resistance to antiretroviral drugs a patient may have, *nres*, at time *t* is calculated as follows:

*nres =*  (o_ZDV x r_ZDV) + (o_DDI x r_DDI) + (o_3TC x r_3TC) + (o_TEN x r_TEN)

+ (o_EFA x r_EFA) + (o_IND x r_IND) + (o_LPR x r_LPR) + (o_DAR x r_DAR)

+ (o_RAL x r_RAL)

where,

o_ZDV = 1 if the patient is on ZDV at time *t*

= 0 if the patient is not on ZDV at time *t*

and recall that:

r_ZDV denotes the level of resistance to ZDV and can take the value 0, 0.25, 0.5, 0.75 or 1.

= 0 if there is no resistance to ZDV at time *t*

= 0.25, 0.5 or 0.75 if there is partial resistance to ZDV at time *t*

= 1 if there is complete resistance to ZDV at time *t*

In other words, *nres* is the total of the level of resistance to each of the antiretroviral drugs the patient is taking at time *t*.

**Comment:** This follows a common approach to reporting drug activity from genotypic (and phenotypic) resistance tests (i.e. this is effectively a genotypic sensitivity score, GSS) (81).

### Superinfection

Individuals already HIV-positive can be infected again by another individual. The MSM transmission model includes this concept of superinfection. This only affects whether an individual acquires transmitted drug resistant virus and therefore subsequently acquires additional resistance mutations.

### Loss of resistance mutations

The loss of mutations considered here refers to those lost in majority virus and not a complete loss, after stopping a regimen and starting another non-cross-resistant regimen. This section considers only the case of a person who has already started ART, and is not about persistence of transmitted mutations (which is currently assumed to be indefinite, except for M184).

Note that if a person was infected with virus with a given mutation, then this mutation is never completely lost.

#### NRTIs

| Mutation lost | Probability of losing mutation | | Conditions |
| --- | --- | --- | --- |
| M184 | 80% | If (M184 mutation is present in majority virus)  and (time since stopping 3TC ≥ 3 months, or no previous use of 3TC) | |
| K65 | 60% | If (K65 mutation is present in majority virus)  and (time since stopping DDI ≥ 3 months, or no previous use of DDI)  and (time since stopping TEN ≥ 3 months, or no previous use of TEN) | |
| L74 | 60% | If (L74 mutation is present in majority virus)  and (time since stopping DDI ≥ 3 months, or no previous use of DDI) | |
| Q151 | 60% | If (Q151 mutation is present in majority virus)  and (time since stopping DDI ≥ 3 months, or no previous use of DDI)  and (time since stopping ZDV ≥ 3 months, or no previous use of ZDV) | |
| TAMS* | 40% | If (# of TAMS ≥ 1)  and (time since stopping ZDV ≥ 3 months, or no previous use of ZDV)  and (time since stopping DDI ≥ 3 months, or no previous use of DDI)  and (time since stopping TEN ≥ 3 months, or no previous use of TEN) | |

When some loses TAMS mutations, the number of TAMS reverts back to the number of TAMS present at the time of infection.

#### NNRTIs

| Mutation lost | Probability of losing mutation | | Conditions |
| --- | --- | --- | --- |
| nn | 20% | If (RTnn mutation is present in majority virus)  and (time since stopping EFA ≥ 3 months, or no previous use of EFA) | |

#### PIs

| Mutation lost | Probability of losing mutation | | Conditions |
| --- | --- | --- | --- |
| V32 | 20% | If (V32 mutation is present in majority virus)  and (time since stopping LPR ≥ 3 months, or no previous use of LPR) | |
| M46 | 20% | If (M46 mutation is present in majority virus)  and (time since stopping IND ≥ 3 months, or no previous use of IND) | |
| I47 | 20% | If (I47 mutation is present in majority virus)  and (time since stopping LPR ≥ 3 months, or no previous use of LPR) | |
| I50V | 20% | If (I50V mutation is present in majority virus)  and (time since stopping DAR ≥ 3 months, or no previous use of DAR) | |
| I54 | 20% | If (I54 mutation is present in majority virus)  and (time since stopping DAR ≥ 3 months, or no previous use of DAR) | |
| L76 | 20% | If (L76 mutation is present in majority virus)  and (time since stopping DAR ≥ 3 months, or no previous use of DAR) | |
| V82 | 20% | If (V82 mutation is present in majority virus)  and (time since stopping IND ≥ 3 months, or no previous use of IND)  and (time since stopping LPR ≥ 3 months, or no previous use of LPR) | |
| I84 | 20% | If (I84 mutation is present in majority virus)  and (time since stopping IND ≥ 3 months, or no previous use of IND)  and (time since stopping DAR ≥ 3 months, or no previous use of DAR) | |

#### Other classes

| Mutation lost | Probability of losing mutation | | Conditions |
| --- | --- | --- | --- |
| IIm | 60% | If (IIm mutation is present in majority virus)  and (time since stopping RAL ≥ 3 months, or no previous use of RAL) | |

**Comment:** This is based on evidence from studies in people interrupting ART (82-87).

### “Regaining” mutations (in majority virus) after restarting ART

Mutations previously present are regained when one of the corresponding drugs is restarted.

## Model fits which are relevant to resistance

### Risk of resistance mutations (and virologic failure) after start of ART (patients starting with PI/r or NNRTI regimen) Observed data from reference (73).

% with at least one resistance mutation (and virologic failure).

| Years from start of HAART | Observed | **Model** |
| --- | --- | --- |
| 1 | 4% | **12%** |
| 2 | 8% | **16%** |
| 3 | 10% | **19%** |
| 4 | 11% | **21%** |
| 5 | 12% | **23%** |
| 6 | 14% | **25%** |
| 7 | 16% | **26%** |
| 8 | 17% | **27%** |

### Risk of resistance mutations after start of ART *Observed data from reference (80).

% with at least one resistance mutation

|  | Years from start of HAART | Observed | **Model** |
| --- | --- | --- | --- |
| M184V mutation | 2 | 6% | **9%** |
| (in those starting with 3TC) | 4 | 13% | **14%** |
|  | 6 | 18% | **17%** |
| TAM | 2 | 4% | **8%** |
| (in those starting with ZDV or d4T) | 4 | 9% | **11%** |
|  | 6 | 13% | **13%** |
| PI mutation | 2 | 3% | **4%** |
| (in those starting with boosted PI regimen) | 4 | 7% | **6%** |
|  | - | - | **-** |
| NNRTI mutation | 2 | 8% | **15%** |
| (in those starting with NNRTI regimen) | 4 | 14% | **20%** |
|  | 6 | 21% | **23%** |

* Observed data are likely to be underestimates as resistance testing is not always performed at virologic failure.

### % with at least one resistance mutation for all three main classes (and virologic failure) Observed data from reference (80).

| Years from start of HAART | Observed | **Model** |
| --- | --- | --- |
| 2 | 1.0% | **0.3%** |
| 4 | 2.7% | **1.6%** |
| 6 | 4.1% | **3.7%** |

### Risk of death after triple class resistance Observed data from reference (88).

% dead by 3 years (for people with triple class resistance up to July 2004)

| Observed | **Model** |
| --- | --- |
| 12% | **21%** |

# Other details

## Interruption of ART

### Treatment interruption

#### Probability of Interruption (due to choice)

The probability of interruption in a given 3 month period whilst a patient is on ART, depends on the ‘underlying tendency to adhere’ (for more details on how adherence is modelled, see section 3) and presence of any current toxicities. It is calculated as follows:

| ‘Underlying tendency to adhere’ | Presence of any current toxicities | Probability of Interruption |
| --- | --- | --- |
| ≥ 0.8 | Yes | 1% |
|  | No | 2% |
| 0.5 – 0.79 | Yes | 1.5% |
|  | No | 3% |
| < 0.5 | Yes | 2% |
|  | No | 4% |

Therefore, the probability of interruption is greater in patients with low ‘underlying tendency to adhere’ and in the presence of current toxicities.

#### Treatment interruption coinciding with patient’s missing clinic visits

There is also a chance that the treatment interruption coincides with patients interrupting visits to the clinic. The probability of this event happening depends on the ‘underlying tendency to adhere’:

- 10% if ‘underlying tendency to adhere’ ≥ 0.8
- 15% if 0.5 ≤ ‘underlying tendency to adhere’ < 0.8
- 20% if ‘underlying tendency to adhere’ < 0.5

#### Clinician aware of treatment interruption

When an individual interrupts treatment, the clinician will not always be aware of the fact that they have stopped treatment. We have incorporated a 30% probability of the clinician not being aware of an incidence of treatment interruption in any 3-month period whilst the individual has stopped treatment. This only affects whether it counts as virologic failure when the patient’s viral load is not suppressed; i.e. if a patient has interrupted ART and the clinic is not aware, then when the viral load shows a rebound, this will be interpreted as virologic failure.

**Comment:** See references (81;89-93).

### Loss to follow-up / Clinic visits

#### Loss to follow-up whilst the patient is OFF ART

‘Loss to follow-up’, implies a period of missing clinic visits, not necessarily indefinite cessation of clinic visits. The probability of being lost to follow-up at any visit whilst the patient is off ART depends on the ‘underlying tendency to adhere’:

- 1% if ‘underlying tendency to adhere’ ≥ 0.8
- 1.5% if 0.5 ≤ ‘underlying tendency to adhere’ < 0.8
- 2% if ‘underlying tendency to adhere’ < 0.5

#### Returning to clinic after loss to follow-up

The probability of returning to clinic visits after loss to follow-up depends on the ‘underlying tendency to adhere’ and whether the person has AIDS.

If the individual developed AIDS in the previous 3-month period, they have an 80% probability of returning to clinic visits. Otherwise, the probability of someone returning to clinic after loss to follow-up is:

- 40% if ‘underlying tendency to adhere’ ≥ 0.8
- 25% if 0.5 ≤ ‘underlying tendency to adhere’ < 0.8
- 13% if ‘underlying tendency to adhere’ < 0.5

If the individual developed CDC B symptoms in the previous 3-month period, these probabilities are increased 5-fold.

### Viral load and CD4 count changes during ART interruption

#### Viral load changes

When an individual interrupts ART, their viral load returns to the maximum viral load (achieved before the time of interruption) in 3 months and adopts natural history changes thereafter (i.e. those in ART-naïve patients, see section 2 for these rates).

#### CD4 count changes

When an individual interrupts ART, their rate of CD4 count decline depends on how long they have interrupted for and also their current viral load. This is summarised in the table below:

| Period of ART interruption | Current viral load (log copies/ml) | Distribution of change in CD4 count (cells/mm3) |
| --- | --- | --- |
| 0-3 months | VL > 5 | Normal (-200,10) |
|  | 4.5 ≤ VL < 5 | Normal (-160,10) |
|  | VL < 4.5 | Normal (-120,10) |
| 3-6 months | VL > 5 | Normal (-100,10) |
|  | 4.5 ≤ VL < 5 | Normal (-90,10) |
|  | VL < 4.5 | Normal (-80,10) |
| 6-9 months | VL > 5 | Normal (-80,10) |
|  | 4.5 ≤ VL < 5 | Normal (-70,10) |
|  | VL < 4.5 | Normal (-60,10) |

If an individual interrupts treatment for more than 3 months, but their CD4 count is over 200 cells/mm3 above their CD4 nadir, then their change in CD4 count over this period of time is the same as for someone who has interrupted for 0-3 months (i.e. first 3 rows in the table).

Once the change in CD4 count has been taken into account, the resulting CD4 count for an individual cannot be less than their CD4 nadir. Their resulting CD4 count will therefore be limited to their CD4 nadir at any time point.

If the individual’s CD4 count reaches the CD4 nadir, then the rate of CD4 count decline adopts natural history changes thereafter (see section 2.4.1 for these rates). However, once the individual interrupts treatment for at least 9 months, their subsequent rate of CD4 count decline also adopts natural history changes, even if the nadir had not been reached by then.

**Comment:** These values are broadly based on evidence from a number of analyses of the effects of ART interruption (89;90;94-102).

### Re-initiation of ART after interruption

The probability of re-starting therapy after treatment interruption (in a person who is visiting the clinic) depends on whether the patient had a CDC B symptom or an AIDS disease in the last 3 months. The probabilities of re-starting are as follows:

- 40% if they had a CDC B symptom
- 25% if they had an AIDS disease

## Toxicities

Toxicities including gastrointestinal symptoms, rash, hepatoxicity, CNS toxicity, lipodystrophy, peripheral neuropathy and nephrolithiasis can occur with certain probability when the individual is on certain specific drugs.

### List of toxicities modelled

| Abbreviation used | Toxicity modelled |
| --- | --- |
| NAU | Nausea |
| DIA | Diarrhoea |
| RAS | Rash |
| CNS | CNS toxicity |
| LIP | Lipodystrophy |
| PEN | Peripheral neuropathy |
| HEP | Hepatoxicity |
| NEPH | Nephrolithiasis |
| OTX | Generic toxicity, with unknown profile |
| ANE | Anaemia |
| HEAD | Headache |
| PANC | Pancreatitis |
| LAC | Lactic acidosis |
| TOX | Any toxicity |

All these variables are binary, i.e. if the individual develops a certain toxicity in a given 3-month period, it takes the value 1, otherwise 0. The variable TOX takes the value 1 if more than one of any of the other toxicities are present and 0 otherwise.

The variable OTX is included so that it is possible to stop new drugs with unknown adverse event profiles.

### Incidence of new current toxicity

All individuals do not have any toxicities at the start of simulation (i.e. point of infection). Summarised below is the percentage probability of developing a new current toxicity in any given 3-month period.

|  | N  A  U | D  I  A | R  A  S | C  N  S | L  I  P | P  E  N | N  E  P  H | O  T  X | A  N  E | H  E  A  D | P  A  N  C | L  A  C |
| --- | --- | --- | --- | --- | --- | --- | --- | --- | --- | --- | --- | --- |
| ZDV | 10 |  |  |  | 1.5 |  |  | 3 | 3 | 10 |  | 0.1 |
| 3TC |  |  |  |  |  |  |  | 3 |  |  |  |  |
| DDI | 10 | 5 |  |  |  | 1 |  | 3 |  |  | 0.1 | 0.1 |
| TEN |  |  |  |  |  |  | 1 | 3 |  |  |  |  |
| EFA |  |  | 3a | 10b |  |  |  | 3 |  |  |  |  |
| IND |  |  |  |  |  |  |  | 3 |  |  |  |  |
| LPR |  | 3 |  |  |  |  |  | 3 |  |  |  |  |
| DAR | 10 |  |  |  |  |  |  | 3 |  |  |  |  |
| CCR |  |  |  |  |  |  |  | 3 |  |  |  |  |

a. If on EFA now, but was not on EFA 6 months ago

b. Have been on current regimen for less than 1 year

The probabilities shown above are independent for each toxicity, only if the values in the table above are different. For example, there is a 10% chance of someone developing NAU if (on ZDV or DDI or DAR).

There is an increased risk of some toxicities in the first year of starting ART. The raised risk is 1.5-fold for NAU, DIA, PEN, OTX, ANE and HEAD.

### Continuation of existing toxicity

Summarised below is the percentage probability of retaining an existing toxicity in any given 3-month period. There are some exceptions (see comment below).

|  | N  A  U | D  I  A | C  N  S | A  N  E | H  E  A  D |
| --- | --- | --- | --- | --- | --- |
| ZDV | 50 |  |  | 20 | 40 |
| 3TC |  |  |  |  |  |
| DDI | 50 | 20 |  |  |  |
| TEN |  |  |  |  |  |
| EFA |  |  | * |  |  |
| IND |  |  |  |  |  |
| LPR |  | 20 |  |  |  |
| DAR | 50 |  |  |  |  |
| CCR |  |  |  |  |  |

* 80% if patient has been on current regimen for less than 1 year. 90% if they have been on current regimen for more than or equal to 1 year.

The interpretation of the above table is as follows. For example, there is a 50% chance of someone with NAU at time *t* still having nausea at *t+1* if (on ZDV or DDI or DAR).

There is an increased risk of some toxicities continuing in the first year of starting ART. The raised risk is 1.5-fold for NAU and DIA, ANE and HEAD.

**Comment:** Once an individual has lipodystrophy, they are modeled to always have lipodystrophy, even if they stop ZDV. Once an individual has peripheral neuropathy, they are modeled to have peripheral neuropathy for as long as stay on DDI (i.e. they will not have it anymore as soon as they stop DDI).

The following toxicities are only given a probability of acquiring it (but not retaining it): rash, hepatoxicity, nephrolithiasis, generic toxicity, pancreatitis, lactic acidosis. The reason for this is because we have modeled it such that once an individual gets one or more of these toxicities, the drug that is causing this toxicity is switched if the toxicity is sufficiently severe and that it will otherwise be resolved.

These probabilities are based broadly on evidence from trials and cohort studies, although there are no common definitions for some conditions which complicate this. Data on toxicities based on drug labels were also used. Further refinement will be possible as more data accumulate.

## Regimen switching

The switching of regimens is dealt by first considering whether the patient should be switching and then considering which antiretrovirals to switch to. The probability of switching depends on whether the switching is being considered because the patient has virologically failed their regimen or because they have developed toxicities to their current regimen.

The choice of drug to switch to is dealt separately in this documentation for ‘pre-2003’ and ‘post-2003’. There is no particular reason other than the two being dealt separately also in the model. After 2003, switching was assumed to be fully based on resistance testing.

### Probability of switching – due to virological failure of regimen

#### Initiation of second line HAART (for people who failed first line and still on first line)

Individuals have a 30% chance of initiating (or switching to) the second line regimen in every 3 month period, after year 2000.

#### Initiation of third line HAART (for people who failed second line and still on second line)

Similar to above, individuals have a 30% chance of initiating (or switching to) the third line regimen in every 3 month period, but only after year 2007.

### Probability of switching – due to toxicity

If toxicity is present then individual drugs may be switched due to toxicity.

The table below summarises the probability of switching (%) due to toxicity in any given 3-month period. Any blanks in the table imply that the particular drug will not be stopped by the corresponding toxicity.

|  | N  A  U | D  I  A | R  A  S | C  N  S | L  I  P | P  E  N | H  E  P | N  E  P  H | O  T  X | A  N  E | H  E  A  D | P  A  N  C | L  A  C |
| --- | --- | --- | --- | --- | --- | --- | --- | --- | --- | --- | --- | --- | --- |
| ZDV | 8 |  |  |  | 1 |  |  |  |  | 5 | 2 |  |  |
| 3TC |  |  |  |  |  |  |  |  |  |  |  |  |  |
| DDI | 5 | 5 |  |  |  | 10 |  |  |  |  |  | 100 |  |
| TEN |  |  |  |  |  |  |  | 100 | 10 |  |  |  |  |
| EFA |  |  | 5 | 0.8 |  |  |  |  |  |  |  |  |  |
| IND | 25 |  |  |  |  |  |  | 40 |  |  |  |  |  |
| LPR | 5 | 5 |  |  |  |  |  |  |  |  |  |  |  |
| DAR |  |  |  |  |  |  |  |  |  |  |  |  |  |
| CCR |  |  |  |  |  |  |  |  |  |  |  |  |  |

## Model fits

### Discontinuation of drugs in initial HAART regimen. Observed data from reference (103).

Time from start of ART to discontinuation of at least one drug in the initial regimen (discontinuation for any reason).

Modelled data for 1996-2001 inclusive.

| Years from start of HAART | Observed | **Model** |
| --- | --- | --- |
| 1 | 30% | **30%** |
| 2 | 45% | **43%** |
| 3 | 62% | **52%** |
| 4 | 73% | **61%** |

Reference List

(1) Kuller LH, Ockene JK, Meilahn E, Wentworth DN, Svendsen KH, Neaton JD. Cigarette-Smoking and Mortality. Preventive Medicine 1991;20(5):638-54.

(2) Mercer CH, Fenton KA, Copas AJ, Wellings K, Erens B, McManus S et al. Increasing prevalence of male homosexual partnerships and practices in Britain 1990-2000: evidence from national probability surveys. AIDS 2004;18(10):1453-8.

(3) Dodds JP, Mercey DE, Parry JV, Johnson AM. Increasing risk behaviour and high levels of undiagnosed HIV infection in a community sample of homosexual men. Sex Transm Infect 2004;80(3):236-40.

(4) Williamson LM, Dodds JP, Mercey DE, Johnson AM, Hart GJ. Increases in HIV-related sexual risk behavior among community samples of gay men in London and Glasgow: How do they compare? J Acquir Immune Defic Syndr 2006;42(2):238-41.

(5) Elford J, Bolding G, Sherr L. High-risk sexual behaviour increases among London gay men between 1998 and 2001: what is the role of HIV optimism? AIDS 2002;16(11):1537-44.

(6) Elford J, Bolding G, Davis M, Sherr L, Hart G. Trends in sexual behaviour among London homosexual men 1998-2003: implications for HIV prevention and sexual health promotion. Sex Transm Infect 2004;80(6):451-4.

(7) Hollingsworth T, Anderson RM, Fraser C. HIV-1 transmission, by stage of infection. J Infect Dis 2008;198(5):687-93.

(8) Cohen MS. Sexually transmitted diseases enhance HIV transmission: no longer a hypothesis. Lancet 1998;351:5-7.

(9) Koot M, Keet IPM, Vos AHV, Degoede REY, Roos MTL, Coutinho RA et al. Prognostic Value of HIV-1 Syncytium-Inducing Phenotype for Rate of CD4+ Cell Depletion and Progression to AIDS. Ann Intern Med 1993;118(9):681-8.

(10) Pantazis N, Touloumi G. Bivariate modelling of longitudinal measurements of two human immunodeficiency type 1 disease progression markers in the presence of informative drop-outs. JRSS C 2005;54:405-23.

(11) Sabin CA, Devereux H, Phillips AN, Hill A, Janossy G, Lee CA et al. Course of viral load throughout HIV-1 infection. J Acquir Immune Defic Syndr 2000;23(2):172-7.

(12) Hubert JB, Burgard M, Dussaix E, Tamalet C, Deveau C, Le Chenadec J et al. Natural history of serum HIV-1 RNA levels in 330 patients with a known date of infection. AIDS 2000;14(2):123-31.

(13) O'Brien TR, Rosenberg PS, Yellin F, Goedert JJ. Longitudinal HIV-1 RNA levels in a cohort of homosexual men. J Acquir Immune Defic Syndr 1998;18(2):155-61.

(14) Henrard DR, Phillips JF, Muenz LR, Blattner WA, Wiesner D, Eyster ME et al. Natural-History of HIV-1 Cell-Free Viremia. JAMA 1995;274(7):554-8.

(15) Lyles RH, Munoz A, Yamashita TE, Bazmi H, Detels R, Rinaldo CR et al. Natural history of human immunodeficiency virus type 1 viremia after seroconversion and proximal to AIDS in a large cohort of homosexual men. J Infect Dis 2000;181(3):872-80.

(16) Touloumi G, Pantazis N, Babiker AG, Walker SA, Katsarou O, Karafoulidou A et al. Differences in HIV RNA levels before the initiation of antiretroviral therapy among 1864 individuals with known HIV-1 seroconversion dates. AIDS 2004;18(12):1697-705.

(17) Mellors JW, Munoz A, Giorgi JV, Margolick JB, Tassoni CJ, Gupta P et al. Plasma viral load and CD4+ lymphocytes as prognostic markers of HIV-1 infection. Ann Intern Med 1997;126(12):946-54.

(18) Touloumi G, Karafoulidou A, Gialeraki A, Katsarou O, Milona I, Kapsimali V et al. Determinants of progression of HIV infection in a Greek hemophilia cohort followed for up to 16 years after seroconversion. J Acquir Immune Defic Syndr 1998;19(1):89-97.

(19) Phillips AN, Elford J, Sabin C, Bofill M, Janossy G, Lee CA. Immunodeficiency and the Risk of Death in HIV-Infection. JAMA 1992;268(19):2662-6.

(20) Lundgren JD, Pedersen C, Clumeck N, Gatell JM, Johnson AM, Ledergerber B et al. Survival differences in European patients with AIDS, 1979-89. BMJ 1994 April 23;308(6936):1068-73.

(21) Wright TL, Hollander H, Pu X, Held MJ, Lipson P, Quan S et al. Hepatitis C in HIV-infected patients with and without AIDS: Prevalence and relationship to patient survival. Hepatology 1994;20(5):1152-5.

(22) Eyster ME, Diamondstone LS, Lien JM, Ehmann WC, Quan S, Goedert JJ. Natural History of Hepatitis C Virus Infection in Multitransfused Hemophiliacs: Effect of Coinfection with Human Immunodeficiency Virus. J Acquir Immune Defic Syndr 1993;6(6).

(23) Soriano V, García-Samaniego J, Valencia E, Rodríguez-Rosado R, Muñoz F, González-Lahoz J. Impact of Chronic Liver Disease Due to Hepatitis Viruses as Cause of Hospital Admission and Death in HIV-Infected Drug Users. European Journal of Epidemiology 1999 January 1;15(1):1-4.

(24) Graham CS, Baden LR, Yu E, Mrus JM, Carnie J, Heeren T et al. Influence of Human Immunodeficiency Virus Infection on the Course of Hepatitis C Virus Infection: A Meta-Analysis. Clin Infect Dis 2001 August 15;33(4):562-9.

(25) Touloumi G, Hatzakis A, Rosenberg PS, O'Brien TR, Goedert JJ. Effects of age at seroconversion and baseline HIV RNA level on the loss of CD4+ cells among persons with hemophilia. AIDS 1998;12(13):1691-7.

(26) The PLATO Collaboration. Predictors of trend in CD4-positive T-cell count and mortality among HIV-1-infected individuals with virological failure to all three antiretroviral-drug classes. Lancet 2004;364(9428):51-62.

(27) CASCADE Collaboration. Short-term risk of AIDS according to current CD4 cell count and viral load in antiretroviral drug-naive individuals and those treated in the monotherapy era. AIDS 2004;18(1):51-8.

(28) Phillips AN, Lee CA, Elford J, Webster A, Janossy G, Timms A et al. More Rapid Progression to AIDS in Older HIV-Infected People - the Role of CD4+ T-Cell Counts. J Acquir Immune Defic Syndr 1991;4(10):970-5.

(29) Mallolas J, Zamora L, Gatell JM, Miro JM, Vernet E, Valls ME et al. Primary Prophylaxis for Pneumocystis-Carinii Pneumonia - A Randomized Trial Comparing Cotrimoxazole, Aerosolized Pentamidine and Dapsone Plus Pyrimethamine. AIDS 1993;7(1):59-64.

(30) Deeks SG, Phillips AN. HIV infection, antiretroviral treatment, ageing, and non-AIDS related morbidity. BMJ 2009;338.

(31) Phillips AN, Neaton J, Lundgren JD. The role of HIV in serious diseases other than AIDS. AIDS 2008;22(18):2409-18.

(32) Frisch M, Biggar RJ, Engels EA, Goedert JJ, for the AIDS-Cancer Match Registry Study Group. Association of Cancer With AIDS-Related Immunosuppression in Adults. JAMA 2001 April 4;285(13):1736-45.

(33) Herida M, Mary-Krause M, Kaphan R+, Cadranel J, Poizot-Martin I, Rabaud C et al. Incidence of Non-AIDS-Defining Cancers Before and During the Highly Active Antiretroviral Therapy Era in a Cohort of Human Immunodeficiency Virus-Infected Patients. J Clin Oncol 2003 September 15;21(18):3447-53.

(34) Maggi P, Quirino T, Ricci E, De Socio GVL, Gadaleta A, Ingrassia F et al. Cardiovascular Risk Assessment in Antiretroviral-Naive HIV Patients. AIDS Patient Care and Stds 2009;23(10):809-13.

(35) Francisci D, Giannini S, Baldelli F, Leone M, Belfiori B, Guglielmini G et al. HIV type 1 infection, and not short-term HAART, induces endothelial dysfunction. AIDS 2009;23(5):589-96.

(36) Lewden C, Chene G, Morlat P, Raffi F, Dupon M, Dellamonica P et al. HIV-infected adults with a CD4 cell count greater than 500 cells/mm3 on long-term combination antiretroviral therapy reach same mortality rates as the general population. J Acquir Immune Defic Syndr 2007;46(1):72-7.

(37) Lodwick RK, Sabin CA, Porter K, Ledergerber B, van Sighem A, Cozzi-Lepri A et al. Death rates in HIV-positive antiretroviral-naive patients with CD4 count greater than 350 cells per mu L in Europe and North America: a pooled cohort observational study. Lancet 2010;376(9738):340-5.

(38) Collaborative Group on AIDS Incubation and HIV Survival and including the CASCADE EU Concerted Action. Time from HIV-1 seroconversion to AIDS and death before widespread use of highly-active antiretroviral therapy: a collaborative re-analysis. Lancet 2000 April 1;355(9210):1131-7.

(39) Lodi S, Phillips AN, Touloumi G, Geskus RB, Meyer L, Thiebaut R et al. Time from Human Immunodeficiency Virus seroconversion to reaching CD4+ cell count thresholds of <200, <350 and <500 cells/mm3: Assessment of need following changes in treatment guidelines. Clin Infect Dis 2011;53(8):817-25.

(40) Dorrucci M, Rezza G, Porter K, Phillips A. Temporal trends in postseroconversion CD4 cell count and HIV load: The Concerted Action on Seroconversion to AIDS and Death in Europe Collaboration, 1985-2002. J Infect Dis 2007;195(4):525-34.

(41) Carrieri MP, Raffi F, Lewden C, Sobel A, Michelet C, Cailleton V et al. Impact of early versus late adherence to highly active antiretroviral therapy on immuno-virological response: a 3-year follow-up study. Antivir Ther 2003;8(6):585-94.

(42) Paterson DL, Swindells S, Mohr J, Brester M, Vergis EN, Squier C et al. Adherence to protease inhibitor therapy and outcomes in patients with HIV infection. Ann Intern Med 2000;133(1):21-30.

(43) Nieuwkerk P, Gisolf E, Sprangers M, Danner S, Prometheus SG. Adherence over 48 weeks in an antiretroviral clinical trial: variable within patients, affected by toxicities and independently predictive of virological response. Antivir Ther 2001;6(2):97-103.

(44) Walsh JC, Mandalia S, Gazzard BG. Responses to a 1 month self-report on adherence to antiretroviral therapy are consistent with electronic data and virological treatment outcome. AIDS 2002;16(2):269-77.

(45) Bangsberg DR, Porco TC, Kagay C, Charlebois ED, Deeks SG, Guzman D et al. Modeling the HIV protease inhibitor adherence-resistance curve by use of empirically derived estimates. J Infect Dis 2004;190(1):162-5.

(46) Bangsberg DR, Moss AR, Deeks SG. Paradoxes of adherence and drug resistance to HIV antiretroviral therapy. J Antimicrob Chem 2004;53(5):696-9.

(47) Bannister WP, Kirk O, Gatell JM, Knysz B, Viard JP, Mens H et al. Regional changes over time in initial virologic response rates to combination antiretroviral therapy across Europe. J Acquir Immune Defic Syndr 2011;42(2):229-37.

(48) Loveday C, Lampe F, Youle M, Tyrer M, Madge S, Sabin CA et al. Potential for transmission of resistant virus: estimation of the proportion of treated people with resistant virus and viral load > 400 cps/mL. 2003.

(49) Lampe FC, Gatell JM, Staszewski S, Johnson MA, Pradier C, Gill MJ et al. Changes over time in risk of initial virological failure of combination antiretroviral therapy - A multicohort analysis, 1996 to 2002. Arch Intern Med 2006;166(5):521-8.

(50) de Béthune MP. Non-nucleoside reverse transcriptase inhibitors (NNRTIs), their discovery, development, and use in the treatment of HIV-1 infection: A review of the last 20 years (1989-2009). Antiviral Res 2010 January;85(1):75-90.

(51) Røge BT, Barfod TS, Kirk O, Katzenstein TL, Obel N, Nielsen H et al. Resistance profiles and adherence at primary virological failure in three different highly active antiretroviral therapy regimens: analysis of failure rates in a randomized study. HIV Med 2004;5(5):344-51.

(52) King M, Brun S, Tschampa J, Moseley J, Kempf D. Exploring the effects of adherence on resistance: use of local linear regression to reveal relationships between adherence and resistance in antiretroviral-naive patients treated with lopinavir/ritonavir or nelfinavir. Antivir Ther 2003;8(3):U103-U104.

(53) De Meyer S, Lathouwers E, Dierynck I, De Paepe E, Van Baelen B, Vangeneugden T et al. Characterization of virologic failure patients on darunavir/ritonavir in treatment-experienced patients. AIDS 2009;23(14):1829-40.

(54) King JR, Wynn H, Brundage R, Acosta EP. Pharmacokinetic enhancement of protease inhibitor therapy. Clin Pharmacokinet 2004;43(5):291-310.

(55) Buss N, Snell P, Bock J, Hsu A, Jorga K. Saquinavir and ritonavir pharmacokinetics following combined ritonavir and saquinavir (soft gelatin capsules) administration. Br J Clin Pharmacol 2001;52(3):255-64.

(56) Cooper CL, Heeswijk RPG, Gallicano K, Cameron DW. A Review of Low-Dose Ritonavir in Protease Inhibitor Combination Therapy. Clin Infect Dis 2003 June 15;36(12):1585-92.

(57) BHIVA Writing Committee on behalf of the BHIVA Executive Committee. British HIV Association (BHIVA) guidelines for the treatment of HIV-infected adults with antiretroviral therapy. HIV Med 2001;2(4).

(58) BHIVA Writing Committee on behalf of the BHIVA Executive Committee. British HIV Association (BHIVA) guidelines for the treatment of HIV-infected adults with antiretroviral therapy. HIV Med 2003;4 Suppl 1.

(59) Staszewski S, Miller V, Sabin C, Schlecht C, Gute P, Stamm S et al. Determinants of sustainable CD4 lymphocyte count increases in response to antiretroviral therapy. AIDS 1999;13(8):951-6.

(60) Ledergerber B, Egger M, Opravil M, Telenti A, Hirschel B, Battegay M et al. Clinical progression and virological failure on highly active antiretroviral therapy in HIV-1 patients: a prospective cohort study. Lancet 1999;353(9156):863-8.

(61) Phillips AN, Staszewski S, Weber R, Kirk O, Francioli P, Miller V et al. HIV viral load response to antiretroviral therapy according to the baseline CD4 cell count and viral load. JAMA 2001;286(20):2560-7.

(62) Staszewski S, Miller V, Sabin C, Carlebach A, Berger AM, Weidmann E et al. Virological response to protease inhibitor therapy in an HIV clinic cohort. AIDS 1999;13(3):367-73.

(63) Gallant JE, Staszewski S, Pozniak AL, DeJesus E, Suleiman JMAH, Miller MD et al. Efficacy and safety of tenofovir DF vs stavuldine in combination therapy in antiretroviral-naive patients - A 3-year randomized trial. JAMA 2004;292(2):191-201.

(64) van Leth F, Phanuphak P, Ruxrungtham K, Baraldi E, Miller S, Gazzard B et al. Comparison of first-line antiretroviral therapy with regimens including nevirapine, efavirenz, or both drugs, plus stavudine and lamivudine: a randomised open-label trial, the 2NN Study. Lancet 2004;363(9417):1253-63.

(65) Mocroft A, Phillips AN, Gatell J, Ledergerber B, Fisher M, Clumeck N et al. Normalisation of CD4 counts in patients with HIV-1 infection and maximum virological suppression who are taking combination antiretroviral therapy: an observational cohort study. Lancet 2007 August 4;370(9585):407-13.

(66) Gras L, Kesselring AM, Griffin JT, van Sighem AI, Fraser C, Ghani AC et al. CD4 cell counts of 800 cells/mm3 or greater after 7 years of highly active antiretroviral therapy are feasible in most patients starting with 350 cells/mm3 or greater. J Acquir Immune Defic Syndr 2007;45(2):183-92.

(67) Bofill M, Janossy G, Lee CA, Macdonaldburns D, Phillips AN, Sabin C et al. Laboratory Control Values for CD4 and CD8 Lymphocytes-T - Implications for HIV-1 Diagnosis. Clin Exp Immunol 1992;88(2):243-52.

(68) Maini MK, Gilson RJC, Chavda N, Gill S, Fakoya A, Ross EJ et al. Reference ranges and sources of variability of CD4 counts in HIV-seronegative women and men. Genitourinary Medicine 1996;72(1):27-31.

(69) Eron JJ, Benoit SL, Jemsek J, Macarthur RD, Santana J, Quinn JB et al. Treatment with Lamivudine, Zidovudine, Or Both in HIV-Positive Patients with 200 to 500 CD4+ Cells Per Cubic Millimeter. N Engl J Med 1995;333(25):1662-9.

(70) Hammer SM, Katzenstein DA, Hughes MD, Gundacker H, Schooley RT, Haubrich RH et al. A trial comparing nucleoside monotherapy with combination therapy in HIV-infected adults with CD4 cell counts from 200 to 500 per cubic millimeter. N Engl J Med 1996;335(15):1081-90.

(71) Egger M, May M, Chene G, Phillips AN, Ledergerber B, Dabis F et al. Prognosis of HIV-1-infected patients starting highly active antiretroviral therapy: a collaborative analysis of prospective studies. Lancet 2002;360(9327):119-29.

(72) Sterne JAC, Hernan MA, Ledergerber B, Tilling K, Weber R, Sendi P et al. Long-term effectiveness of potent antiretroviral therapy in preventing AIDS and death: a prospective cohort study. Lancet 2005;366(9483):378-84.

(73) The UK Collaborative Group on HIV Drug Resistance and UK CHIC Study Group. Long Term Probability of Detecting Drug-Resistant HIV in Treatment-Naive Patients Initiating Combination Antiretroviral Therapy. Clin Infect Dis 2010;50(9):1275-85.

(74) Smith CJ, Phillips AN, Hill T, Fisher M, Gazzard B, Porter K et al. The rate of viral rebound after attainment of an HIV load < 50 copies/mL according to specific antiretroviral drugs in use: Results from a multicenter cohort study. J Infect Dis 2005;192(8):1387-97.

(75) The Pursuing Later Treatment Options II (PLATO II) Project Team for the Collaboration of Observational HIV Epidemiological Research Europe (COHERE). Triple-Class Virologic Failure in HIV-Infected Patients Undergoing Antiretroviral Therapy for Up to 10 Years. Arch Intern Med 2010;170(5):410-9.

(76) Johnson VA, Calvez V, Günthard HF, Paredes R, Pillay D, Shafer RW et al. 2011 Update of the Drug Resistance Mutations in HIV-1. Top Antivir Med 2011;19(4):156-64.

(77) Corvasce S, Violin M, Romano L, Razzolini F, Vicenti I, Galli A et al. Evidence of differential selection of HIV-1 variants carrying drug-resistant mutations in seroconverters. Antivir Ther 2006;11(3):329-34.

(78) Turner D, Brenner B, Routy JP, Moisi D, Rosberger Z, Roger M et al. Diminished representation of HIV-1 variants containing select drug resistance-conferring mutations in primary HIV-1 infection. Jaids-Journal of Acquired Immune Deficiency Syndromes 2004;37(5):1627-31.

(79) Harrigan PR, Hogg RS, Dong WWY, Yip B, Wynhoven B, Woodward J et al. Predictors of HIV drug-resistance mutations in a large antiretroviral-naive cohort initiating triple antiretroviral therapy. J Infect Dis 2005;191(3):339-47.

(80) The UK Collaborative Group on HIV Drug Resistance and UK CHIC Study Group. Long term probability of detection of HIV-1 drug resistance after starting antiretroviral therapy in routine clinical practice. AIDS 2005;19(5):487-94.

(81) DeGruttola V, Dix L, D'Aquila R, Holder D, Phillips A, it-Khaled M et al. The relation between baseline HIV drug resistance and response to antiretroviral therapy: re-analysis of retrospective and prospective studies using a standardized data analysis plan. Antivir Ther 2000;5(1):41-8.

(82) Devereux HL, Emery VC, Johnson MA, Loveday C. Replicative fitness in vivo of HIV-1 variants with multiple drug resistance-associated mutations. J Med Virol 2001;65(2):218-24.

(83) Birk M, Svedhem V, Sonnerborg A. Kinetics of HIV-1 RNA and resistance-associated mutations after cessation of antiretroviral combination therapy. AIDS 2001;15(11):1359-68.

(84) Deeks SG, Grant RM, Wrin T, Paxinos EE, Liegler T, Hoh R et al. Persistence of drug-resistant HIV-1 after a structured treatment interruption and its impact on treatment response. AIDS 2003;17(3):361-70.

(85) Walter H, Low P, Harrer T, Schmitt M, Schwingel E, Tschochner M et al. No evidence for persistence of multidrug-resistant viral strains after a 7-month treatment interruption in an HIV-1-infected individual. J Acquir Immune Defic Syndr 2002;31(2):137-46.

(86) Hance AJ, Lemiale V, Izopet J, Lecossier D, Joly V, Massip P et al. Changes in human immunodeficiency virus type 1 populations after treatment interruption in patients failing antiretroviral therapy. J Virol 2001;75(14):6410-7.

(87) Tarwater PM, Parish M, Gallant JE. Prolonged treatment interruption after immunologic response to highly active antiretroviral therapy. Clin Infect Dis 2003;37(11):1541-8.

(88) Grover D, Copas A, Green H, Edwards SG, Dunn DT, Sabin C et al. What is the risk of mortality following diagnosis of multidrug-resistant HIV-1? J Antimicrob Chem 2008;61(3):705-13.

(89) Li XH, Margolick JB, Conover CS, Badri S, Riddler SA, Witt MD et al. Interruption and discontinuation of highly active antiretroviral therapy in the multicenter AIDS cohort study. J Acquir Immune Defic Syndr 2005;38(3):320-8.

(90) d'Arminio Monforte A, Cozzi-Lepri A, Phillips A, De Luca A, Murri R, Mussini C et al. Interruption of highly active antiretroviral therapy in HIV clinical practice - Results from the Italian cohort of antiretroviral-naive patients. J Acquir Immune Defic Syndr 2005;38(4):407-16.

(91) Mocroft A, Youle M, Moore A, Sabin CA, Madge S, Lepri AC et al. Reasons for modification and discontinuation of antiretrovirals: results from a single treatment centre. AIDS 2001;15(2):185-94.

(92) Concorde Coordinating Committee. Concorde: MRC/ANRS randomised double-blind controlled trial of immediate and deferred zidovudine in symptom-free HIV infection. The Lancet 1994 April 9;343(8902):871-81.

(93) Cotton P. Use of antiretroviral drugs in HIV disease declines following preliminary results from Concorde trial. JAMA 1994 February 16;271(7):488-9.

(94) Skiest DJ, Morrow P, Allen B, McKinsey J, Crosby C, Foster B et al. It is safe to stop antiretroviral therapy in patients with preantiretroviral CD4 cell counts > 250 cells/mL. J Acquir Immune Defic Syndr 2004;37(3):1351-7.

(95) Youle M, Janossy G, Turnbull W, Tilling R, Loveday C, Mocroft A et al. Changes in CD4 lymphocyte counts after interruption of therapy in patients with viral failure on protease inhibitor-containing regimens. AIDS 2000;14(12):1717-20.

(96) Lawrence J, Mayers DL, Hullsiek KH, Collins G, Abrams DI, Reisler RB et al. Structured treatment interruption in patients with multidrug-resistant human immunodeficiency virus. N Engl J Med 2003;349(9):837-46.

(97) Tebas P, Henry K, Mondy K, Deeks S, Valdez H, Cohen C et al. Effect of prolonged discontinuation of successful antiretroviral therapy on CD4+ T cell decline in human immunodeficiency virus-infected patients: Implications for intermittent therapeutic strategies. J Infect Dis 2002;186(6):851-4.

(98) Fischer M, Hafner R, Schneider C, Trkola A, Joos B, Joller H et al. HIV RNA in plasma rebounds within days during structured treatment interruptions. AIDS 2003;17(2):195-9.

(99) Boschi A, Tinelli C, Ortolani P, Moscatelli G, Morigi G, Arlotti M. CD4+cell-count-guided treatment interruptions in chronic HIV-infected patients with good response to highly active antiretroviral therapy. AIDS 2004;18(18):2381-9.

(100) Achenbach CJ, Till M, Palella FJ, Knoll MD, Terp SM, Kalnins AU et al. Extended antiretroviral treatment interruption in HIV-infected patients with long-term suppression of plasma HIV RNA. HIV Med 2005;6(1):7-12.

(101) Thiebaut R, Pellegrin I, Chene G, Viallard JF, Fleury H, Moreau JF et al. Immunological markers after long-term treatment interruption in chronically HIV-1 infected patients with CD4 cell count above 400x10(6) cells/I. AIDS 2005;19(1):53-61.

(102) Wit FWNM, Blanckenberg DH, Brinkman K, Prins JM, van der Ende ME, Schneider MME et al. Safety of long-term interruption of successful antiretroviral therapy: the ATHENA cohort study. AIDS 2005;19(3):345-8.

(103) Mocroft A, Phillips AN, Soriano V, Rockstroh J, Blaxhult A, Katlama C et al. Reasons for stopping antiretrovirals used in an initial highly active antiretroviral regimen: Increased incidence of stopping due to toxicity or patient/physician choice in patients with hepatitis C coinfection. AIDS Res Hum Retroviruses 2005;21(9):743-52.
