# Supplementary material for: Increased HIV Incidence in Men Who Have Sex with Men Despite High Levels of ART-Induced Viral Suppression: Analysis of an Extensively Documented Epidemic
Source: PLoS One. 2013 Feb 15;8(2):e55312. doi: 10.1371/journal.pone.0055312 (PMC3574102; doi:10.1371/journal.pone.0055312)
Supplement: Supporting Information S2 — Supplementary Analysis Methods and Results. (DOC) [file pone.0055312.s002.doc]

**Supporting information S2**

**Increased HIV incidence in men who have sex with men despite high levels of ART use: analysis of an extensively documented epidemic**

**Supplementary Analysis Methods and Results**

**Analysis Methods**

The parameters relating to sexual risk behaviour, transmission and testing and the distributions from which potential values were sampled are shown in Supp Table 1. Seven parameters determine the sexual behaviour: relative average sexual behaviour with short-term partners (μ_7_) the skewness in the distribution of number of short-term condomless sex partners (μ_2_ and μ_3_, with the former determining the most extreme numbers of short-term sexual partners), the rate with which new long term condomless sex partnerships are formed (μ_4_), and the proportions of men who have a lifetime reduced likelihood of short term condomless sex partners (μ_6_). A correlation is induced by the sampling of the parameter μ_7_ in order to provide a focus on parameter space most likely to give low values of the overall fit. For example, if the sampling of μ_1-_μ_5_ is such that values chosen are at the higher end of the distribution and μ_6_ is at the lower end of the distribution then the simulation run will produce an epidemic which is too large, unless there is some compensation when selecting the value of μ_7_. To indicate what these distributions mean in terms of the proportion of men with a condomless anal sex partner (short or long term) in the past year, they are such that the distribution ranges from 0.10 to 0.92 (5%-95% range 0.22-0.50) in 1995.

Parameters relating to transmission are the transmission rate per 3 months per short term condomless partner with HIV (μ_1_) with a distribution representing the level of uncertainty and the fold higher risk of transmisison per 3 months from a long term partner compared with short term (due to more sex acts - μ_5_). As illustrated in Supp Fig 1, we specified changes in relative sexual risk behaviour (compared with that in 1980) from 1980 to 1998, with a decline in the early 1980's (as was observed^1^), followed by a prolonged period to 1998 of reduced risk behaviour. There is evidence of significant increases in risk behaviour in the late 1990's ^2-6^, and likewise there was likely an increase in testing in 2001 with introduction of opt-out testing in GUM clinics ^7^. We therefore parameterized changes in risk behaviour and testing assuming a step increase in 1998 for risk behaviour followed by a linear change, and a linear change in underlying testing rate from 2001. Suppl Table 1 shows the distributions from which we sampled potential values for the parameters. Parameters relating to natural history of HIV and the effect of ART were fixed, using values which have previously been shown to give a close fit to observed data ^8,9^.

Model outputs were formally compared with data obtained as part of HIV surveillance activities carried out by the HPA (numbers seen for care for HIV in 2005 and 2010, cumulative numbers testing positive for HIV in time periods 1984-2002, 2003-2005, 2006-2008, 2009-2010, estimated number of people living with HIV aged 15-59 in 2008, median CD4 count at diagnosis in 2005, 2008 and 2010, the percent diagnosed within 6 months of infection in 2009/2010 ^10^, the proportion of MSM reporting having tested for HIV in the past year in 2008 and the proportion of men having at least one condomless sex partner in the past year in 2000^11^. Supp Table 2 shows the observed values of these data. For each data item the fit was calculated as |observed value - model output value| / observed value. We divided here by the observed rather than the model output value as this provided greater stability. The overall fit was calculated as the sum of the fit to each of the data items with the weights shown in Supp Table 2. The weights were based on a subjective judgement and chosen to reflect prior considerations of the importance placed that on the model fitting the various different data items and the confidence in the data. For the proportion of men tested in the past year data from the Gay Men's Sex Survey suggest a value > 30%, while number of tests performed in MSM suggest a figure closer to 10% and we used a value of 15%, with the low weighting reflecting the uncertainty over the data. Although not part of the formal fitting, we also compared with estimates of the number of men living with HIV, using the MPES approach, based on prevalence survey data ^12^.

The model was run 10,000 times, independently varying at random these parameter values with distribution as shown in Supp Table 2. For parameter sets with fit value < 2 we repeated runs multiple times and kept only parameter sets for which the fit value was consistently < 2. To generate uncertainty bounds we first excluded any runs for which the value for the overall fit value was > 2 then the median and 90% range (5%-95% centiles) over remaining runs were plotted. These parameter sets were used when considering counter-factual scenarios in which ART was not used, condoms ceased to be used, there were higher levels or testing and where all people were treated with ART at diagnosis. Uncertainty bounds for the counter-factual scenarios are based on the 90% range of all runs in the counter-factual scenario, excluding runs for which the fit value for the cumulative number with HIV diagnosed by 1998 was > 0.15..

**Results of model fitting**

From the 10,000 runs, 262 parameter sets gave a fit value < 2. A comparison of model outputs for these 262 runs with a range of observed data is shown in Fig 1 of the main paper. Supp Figure 2 shows the fit of this model to other data, and further model outputs. For these 262 parameter sets resulting in good model fit the distribution of the parameter value indexing change in condomless sex with short term partners after 1998, β_r_, had a median 0.012 (90% range 0.002 - 0.022), supporting the fact there was an increase in condomless sex. After re-running multiple times using these parameter sets we restricted to 58 parameter sets for which the fit value was consistently < 2 for use in assessing counter-factual scenarios. Uncertainty bounds were each based on over 150 runs in which parameter sets were sampled from the 58 sets.

**Supp Fig. 1.** Parameterization of changes in average sexual behaviour and rate of testing.


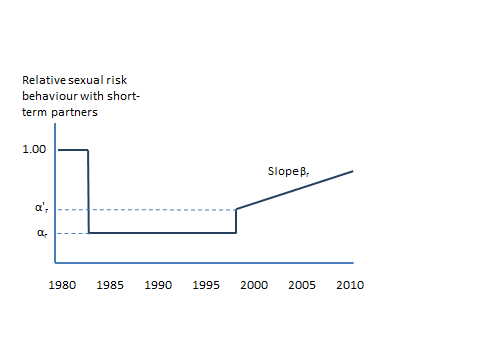


(a)


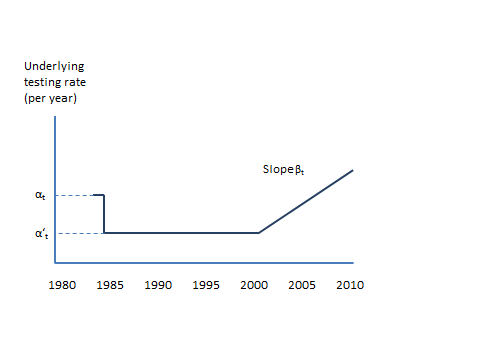


(b)

**Supp Fig 2** - Further model outputs (continuation of Figure 1 in main manuscript)


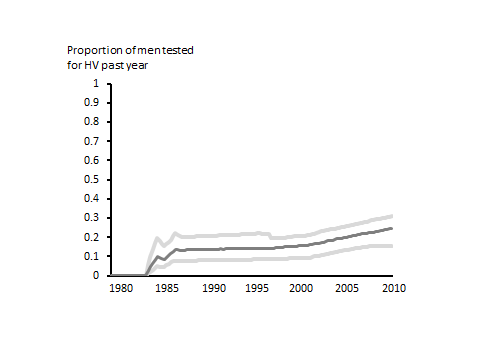
(a) Proportion of all HIV negative MSM tested in the past year,


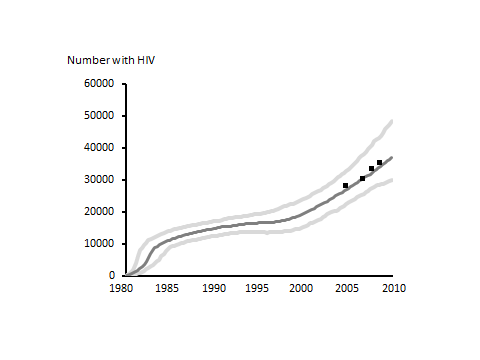
(b) Number of people living with HIV aged 15-60. "Data" are estimates from MPES method which uses data from HIV prevalence surveys ^1, 41^,

(c) Proportion diagnosed within 6 months of infection (HPA). Data points from HPA ^1^,


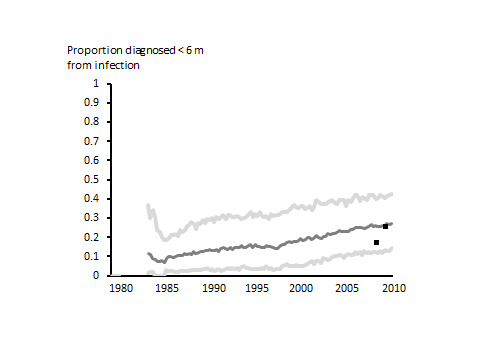


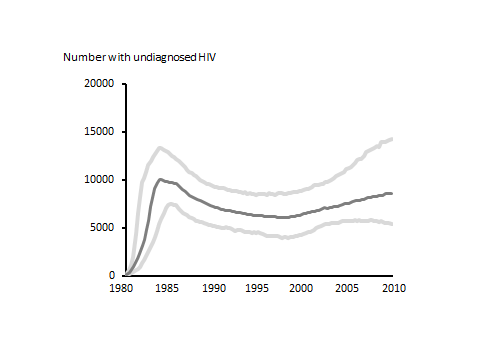


(d) Number of men with undiagnosed HIV.


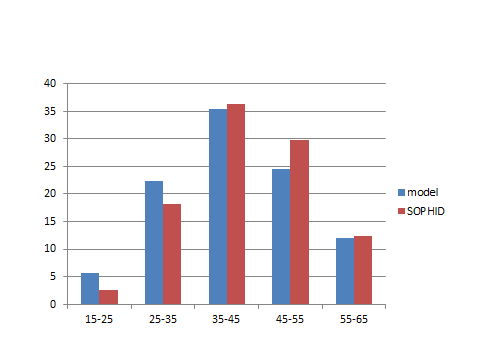
(e) age distribution for men seen for care in 2010 (observed data from SOPHID ^10^)


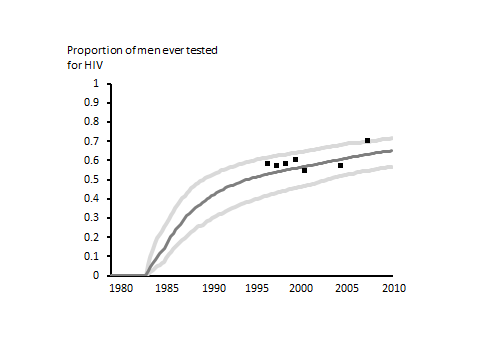


(f) Proportion ever tested for HIV. Observed data (black squares) from Gay Men's Sex Survey ^6^.


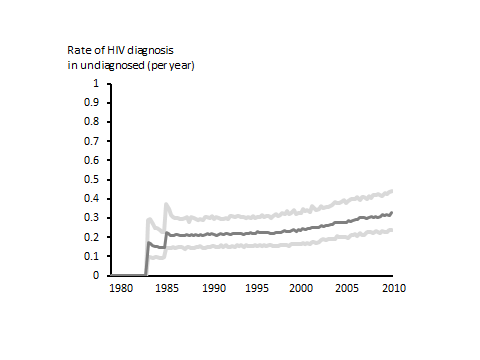
(g) Rate of HIV diagnosis in those with undiagnosed HIV


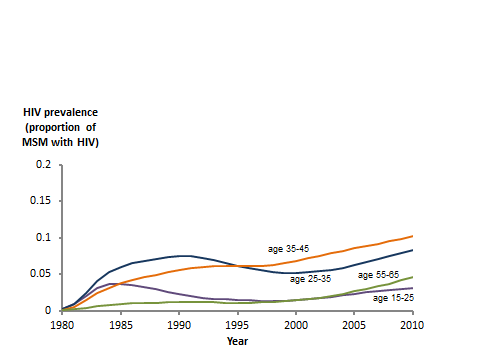
(h) HIV prevalence by age group


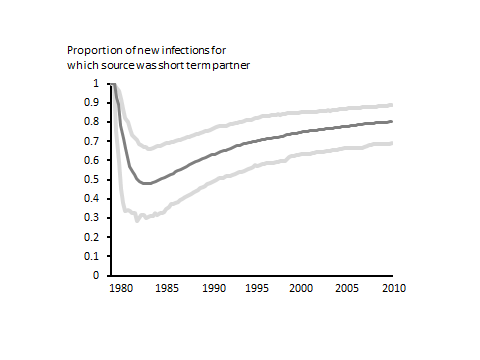


(i) Proportion of men infected by a short-term partner


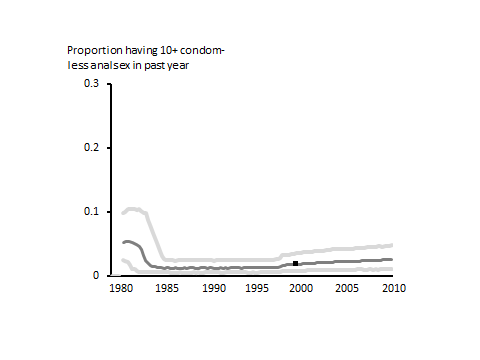
(j) Proportion of men reporting > 10 condomless anal intercourse partners in past year. Data point from 2010 is from NATSAL (Mercer C, personal communication)


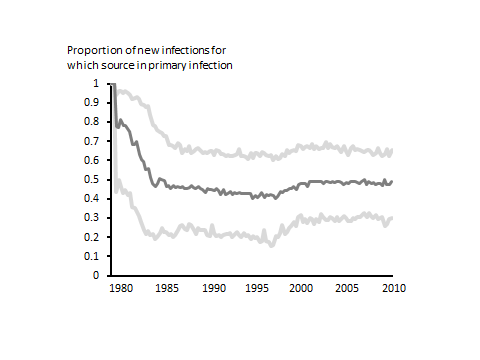


(k) Proportion of men infected by a man in primary infection


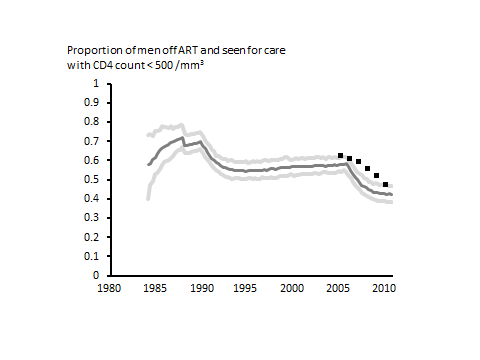
(l) Proportion of men under care and not on ART with CD4 count < 500. Data (black squares) from SOPHID ^10^.


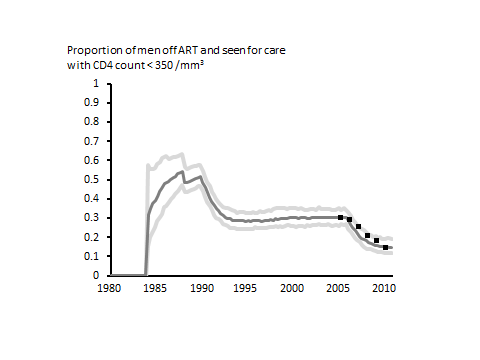
(m) Proportion of men under care and not on ART with CD4 count < 350. Data (black squares) from SOPHID ^10^.

(n) Proportion of men under care and not on ART with CD4 count < 200. Data (black squares) from SOPHID ^10^.


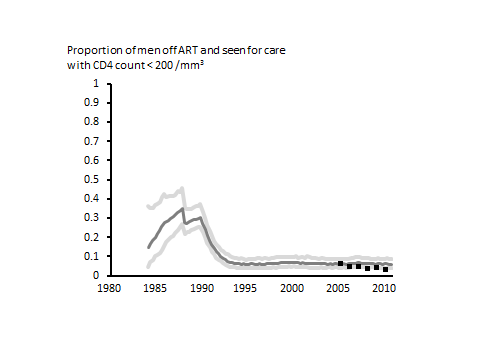


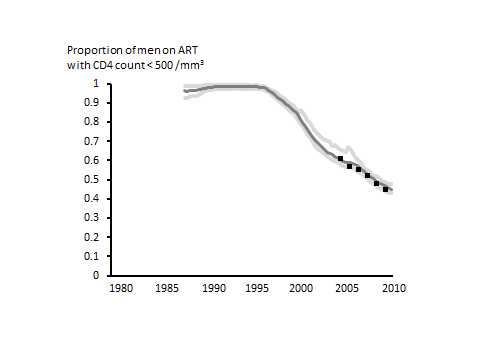


(o) Proportion of men on ART with CD4 count < 500. Data (black squares) from SOPHID ^10^.


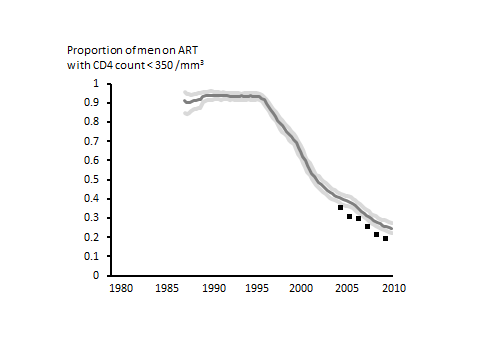


(p) Proportion of men on ART with CD4 count < 350. Data (black squares) from SOPHID ^10^.

(q) Proportion of men on ART with CD4 count < 200. Data (black squares) from SOPHID ^10^.


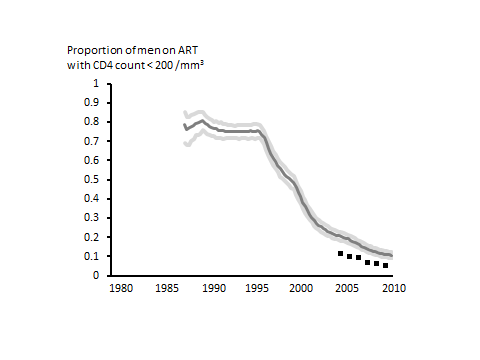


**Supplementary Table 1.** Parameter values and distributions reflecting uncertainty in parameter values**^+^**.

**Parameter Value / distribution Basis for choice**

-----------------------------------------------------------------------------------------------------------------------------------------

**Parameters relating to risk behaviour and**

**transmission**

Rate of transmission through anal sex per 3 months Refs 13-17

Short term condomless sex partner

primary infection 0.100

VL > 5.7 log 0.050

4.7 < VL < 5.7 log 0.030

3.7 < VL < 4.7 log 0.015

2.7 < VL < 3.7 log 0.005

VL < 2.7 log 0.0001

Fold change in infectivity for given VL (μ_1_) exp (Normal(0, 0.25)

Value of multiplicative factor determining 7 x exp (Normal(0, 0.25) 11-18, numbers of partners for those in highest (incl personal

new partner group (see supplementary methods comm from

describing model) (μ_2_) authors)

Value of and fold change in multiplicative factor 3.5 x exp (Normal(0, 0.25) as above determining numbers of partners for those in second

highest new partner group (see supplementary

methods describing model details) (μ_3_)

Probability of new long term condomless sex partner 0.06 x exp (Normal(0, 0.25) Assumption

(per 3 months) (μ_4_)

Fold higher risk of transmission to long term 4 x exp (Normal(0, 0.25) Assumption

condomless sex partner compared with short term & preliminary

(due to a greater number of sex acts) (μ_5_) fitting~

Proportion of individuals with a 50% (75%) reduced 0.4 x exp (Normal(0, 0.25) Assumption

lifetime likelihood of short term partners (μ_6_) (above value / 2) & preliminary

fitting~

Factor determining median number of new partners ( μ_6_ / μ_1_.μ_2_.μ_3._μ_4_.μ_5_ ) Preliminary

(μ_7_) * x exp (Normal(0,0.10) fitting~

Fold change in rate of transmission rate when viral exp (Normal(0, 2.0) Perceived

load undetectable for short term partners uncertainty

(per 3 months)

α_r_ (see Supp Fig 1)^ 0.27 x exp (Normal(0, 0.25)) Ref 1, Preliminary

fitting~

α'_r_ (see Supp Fig 1) 0.35 x exp (Normal(0, 0.25)) Preliminary

fitting~

β_r_ (see Supp Fig 1) Normal (0.012, 0.006) Refs 2-6

Proportion of men who substantially reduce number

of new partners (by 90%) after HIV diagnosis Beta (7,7) Ref 18, 19

Proportion of men who substantially reduce probability Beta (5,20) Ref 19

of condomless sex with long term negative partner

(by 90%) after HIV diagnosis

**Parameters related to testing and diagnosis**

α_t_ (see Supp Fig 1) 0.12 x exp (Normal(0, 0.25)) Preliminary

fitting~

α'_t_ (see Supp Fig 1) 0.28 x exp (Normal(0, 0.25)) Preliminary

fitting~

β_t_ (see Supp Fig 1) Normal (0.005, 0.003) Ref 7

Fold reduction in probability of testing if no Normal (3.5, 1.0) Assumption,

condomless sex partner in past year based on preliminary

fitting~

Fold increase in probability of testing if Normal (3.0, 1.0) Assumption,

at least one condomless sex partner in past year based on

preliminary fitting~

Proportion with reluctance to test 0.50 x exp (Normal(0, 0.25) Refs 6,11

(4-fold reduced testing rate)

Proportion not testing (if no AIDS) 0.25 x exp (Normal(0, 0.25) Ref 6

----------------------------------------------------------------------------------------------------------------------------------

+ Further details on parameter values is given in Supplementary Model Details. Distributions here reflect perceived level of uncertainty, which are subjective. Parameter values are derived directly from the cited literature in some cases but in most the cited literature did not present estimates of parameter values exactly corresponding to parameterization of the model and was thus used to inform the presence and approximate magnitude of relationships but not to directly provide the parameter value used. Nonetheless, our inferences are based on sets of parameter values which result in a good fit to the data, as illustrated in Fig 1 and Suppl Fig 2. The resulting fit of the model to data on HIV natural history and the effect of ART is shown in supplementary model details.

***** the correlation induced by the sampling of this parameter is to provide a focus on parameter space most likely to give low values of the overall fit. For example, if the sampling of μ_1-_μ_5_ is such that values chosen are at the higher end of the distribution and μ_6_ is at the lower end of the distribution then the simulation run will produce an epidemic which is too large, unless there is some compensation when selecting the value of this parameter.

^ The exact timing (between 1983 and 1985) of the reduction in risk behaviour in the 1980s is dependent on the number of people infected such that the reduction occurs when 7000 men have been infected. This is due to the stochastic run-to-run variability (for the same parameter value set).

** parameters relating to natural history of HIV and the effect of ART were fixed, using values which have previously been shown to give a close fit to observed data ^8,9^

**~** preliminary fitting refers to the informal stage of model building from our highly parameterized starting model in which the structure is derived, based on a decision to hold certain parameters fixed and thus form part of the structure, and the plausible range of values for other parameters is ascertained. This process of how to simplify a complex model into a simpler one to address a given question is in many ways equivalent to the process of building a new model to address a given question.

**Supplementary Table 2.**

Data item used in fit+ Weight Observed

Value

---------------------------------------------------------------------------------------------------------------------

numbers seen for care for HIV in 2005 1.0 20041

2010 1.0 29647

cumulative numbers testing positive for HIV in time periods

1984-1998 1.0 23430

1999-2002 1.0 6753

2003-2005 1.0 7169

2006-2008 1.0 8030

2009-2010 1.0 5276

median CD4 count at diagnosis in 2005 1.0 390

median CD4 count at diagnosis in 2008 1.0 405

median CD4 count at diagnosis in 2010 1.0 415

percent diagnosed within 6 months of infection in 2009/2010) 0.5 0.21

proportion of MSM reporting having tested for HIV in the past

year in 2008*) 0.2 0.15*

percent reporting condomless anal intercourse in past year 0.2 0.37**

---------------------------------------------------------------------------------------------------------------------

+ note that we compared the outputs of the final model with a larger array of data (see fig 1 in main manuscript and supp Fig 2). All data from HPA ^10^ except where stated.

* data from Gay Men's sex Survey ^6^ suggest higher values (> 30%), while number of tests performed in MSM suggest a figure closer to 10%. The low weighting reflects the uncertainty over the data.

** NATSAL 2000 ^11^

**References for Supplementary Methods and Results**

1. Carne CA, Weller IVD, Johnson AM, Loveday C, Pearce F, et al. Prevalence of antibodies to HIV, gonorrhoea rates, and changed sexual behaviour in homosexual men in London. Lancet 1987; 656-658.
2. Dodds JP, Mercey DE, Parry JV, Johnson AM. Increasing risk behaviour and high levels of undiagnosed HIV infection in a community sample of homosexual men. Sex Transm Infect 2004; 80:236-240.
3. Williamson LM, Dodds JP, Mercey DE, et al. Increases in HIV-related sexual risk behaviour among community samples of gay men in London and Glasgow: how do they compare? J Acquir Immune Defic Syndr 2006;42:238–41.
4. Elford J, Bolding G, Sherr L. High-risk sexual behaviour increases among London gay men between 1998 and 2001: what is the role of HIV optimism? AIDS 2002, 16:1537–1544
5. Elford J, Bolding G, Davis M, Sherr L, Hart G. Trends in sexual behaviour among London homosexual men 1998-2003: implications for HIV prevention and sexual health promotion. Sex Transm Infect 2004; 80:451-454.
6. http://www.sigmaresearch.org.uk/reports/
7. Dougan S, Elford J, Chadborn TR, Brown AE, Roy K, Murphy G, et al. Does the recent increase in HIV diagnoses among men who have sex with men in the UK reflect a rise in HIV incidence or increased uptake of HIV testing. Sex Transm Infect 2007; 83:120-126.
8. Phillips AN, Sabin C, Pillay D and Lundgren JD. HIV in the UK 1980-2006: reconstruction using a model of HIV infection and the effect of antiretroviral therapy. HIV Med 2007;8:536-546.
9. Nakagawa F, Lodwick RK, Smith CJ, Smith R, Cambiano V, Lundgren JD, Delpech V, Phillips AN. Projected life expectancy of people with HIV according to timing of diagnosis. AIDS 26(3): 335-343; Jan 2012
10. http://www.hpa.org.uk/Topics/InfectiousDiseases/InfectionsAZ/HIV/
11. Mercer CH, Fenton KA, Copas AJ, Wellings K, Erens B, McManus S, et al. Increasing prevalence of male homosexual partnerships and practices in Britain 1990-2000: evidence from national probability surveys. AIDS 2004; 18:1453-1458.
12. Presanis AM, Gill ON, Chadborn TR, Hill C, Hope V, et al. Insights into the rise in HIV infections, 2001 to 2008: a Bayesian synthesis of prevalence evidence. AIDS 2010; 24:2849–2858.
13. Quinn TC, Wawer MJ, Sewankambo N, Serwadda D, Li C, Wabwire-Mangen F, et al. Viral load and heterosexual transmission of human immunodeficiency virus type 1. Rakai Project Study Group. N Engl J Med 2000 Mar 30;342(13):921-9.
14. Jin F, Jansson J, Law M, Prestage GP, Zablotska I, Imrie JCG et al. Per-contact probability of HIV transmission in homosexual men in Sydney in the era of HAART. AIDS 2010; 24*:*907–913.
15. Hollingsworth TD, Anderson RM, Fraser C. HIV-1 transmission, by stage of infection. J Infect Dis. 2008; 198(5):687–693.
16. Vittinghoff E, Douglas J, Judson F, McKirnan D, MacQueen K, Buchbinder SP. Per-contact risk of human immunodeficiency virus transmission between male sexual partners. Am J Epidemiol 1999;150:306–11.
17. Baggaley RF,White RG, Boily MCHIV transmission risk through anal intercourse:systematic review, meta-analysis and implications for HIV prevention. International Journal of Epidemiology 2010;39:1048–1063.
18. Williamson LM, Dodds JP, Mercey DE, Hart GJ, Johnson AM. Sexual risk behaviour and knowledge of HIV status among community samples of gay men in the UK. AIDS 2008, 22*:*1063–1070.
19. Marks G, Crepaz N, Senterfitt JW, Janssen R. Meta-analysis of high risk sexual behaviour in persons aware and unaware that they are infected with the virus in the United States: implications for HIV prevention programmes. JAIDS 2005; 39:446-453.
